# Numerical error analysis of the ICZT algorithm for chirp contours on the unit circle

**DOI:** 10.1038/s41598-020-60878-7

**Published:** 2020-03-17

**Authors:** Vladimir Sukhoy, Alexander Stoytchev

**Affiliations:** Department of Electrical and Computer Engineering, Iowa State University, Ames, IA 50011 USA

**Keywords:** Electrical and electronic engineering, Mathematics and computing

## Abstract

This paper shows that the inverse chirp z-transform (ICZT), which generalizes the inverse fast Fourier transform (IFFT) off the unit circle in the complex plane, can also be used with chirp contours that perform partial or multiple revolutions on the unit circle. This is done as a special case of the ICZT, which in algorithmic form has the same computational complexity as the IFFT, i.e., *O*(*n* log *n*). Here we evaluate the ICZT algorithm for chirp contours on the unit circle and show that it is numerically accurate for large areas of the parameter space. The numerical error in this case depends on the polar angle between two adjacent contour points. More specifically, the error profile for a transform of size *n* is determined by the elements of the Farey sequence of order *n* − 1. Furthermore, this generalization allows the use of non-orthogonal frequency components, thus lifting one of the main restrictions of the IFFT.

## Introduction

The *Inverse Chirp Z-Transform* (ICZT) is a generalization of the *Inverse Fast Fourier Transform* (IFFT), which is one of the most popular and useful algorithms^1,2^. The sampling points used by the ICZT are distributed along a logarithmic spiral contour in the complex plane. The shape of this contour is determined by the complex parameters *A* and *W* such that the *k*-th contour point is equal to *AW*^−*k*^, where *k* is a zero-based index (see Supplementary Section S1).

This paper studies the properties of the ICZT for the special case when the magnitudes of *A* and *W* are equal to 1, which restricts the contour to lie on the unit circle. Unlike the IFFT, the ICZT can work with a contour that performs a partial revolution, a full revolution, or more than one revolution. The effect of this generalization is that the frequency components specified by the sampling points are no longer restricted to be harmonically related or orthogonal. Lifting this restriction makes it possible to use the spectrum more efficiently.

Many applications require both *signal analysis* and *signal synthesis*. Traditionally, these tasks have been performed with the FFT and IFFT algorithms that were published in 1965^3^. Both algorithms run in *O*(*n* log *n*) time, which makes them fast and practical. The *Chirp Z-Transform* (CZT), which generalizes the *Fast Fourier Transform* (FFT) and also runs in *O*(*n* log *n*) time, was discovered in 1969^4–9^. The inverse algorithm, however, remained elusive for the next 50 years. The ICZT algorithm also runs in *O*(*n* log *n*) time^10^, where *n* is the size of the transform. This enables applications in which the CZT is paired with the ICZT similarly to how the FFT is often paired with the IFFT. Application domains that could benefit from this include signal processing, electronics, medical imaging, radar, sonar, wireless communications, and others.

**Figure 1:**
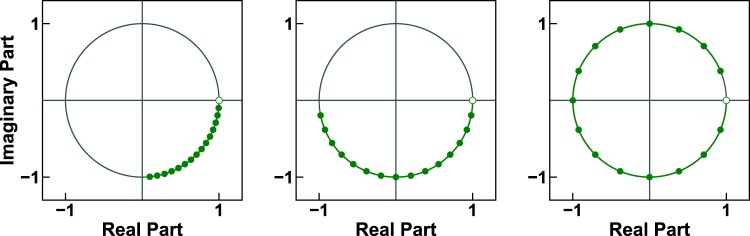
Three chirp contours on the unit circle, each with 16 points. The angular interval between any two adjacent points is: 90°/16, 180°/16,  and 360°/16. The starting point of each contour is indicated with an unfilled circle.

  Figure 1 shows three examples of 16-point chirp contours that lie on the unit circle. The spacing between the 16 points is different in each plot. From left to right, the angular interval between neighboring points is equal to: 5.625°, 11.25°, and 22.5°. The last contour corresponds to the FFT after reordering the output vector elements (see Supplementary Sections S1 and S2).

**Figure 2:**
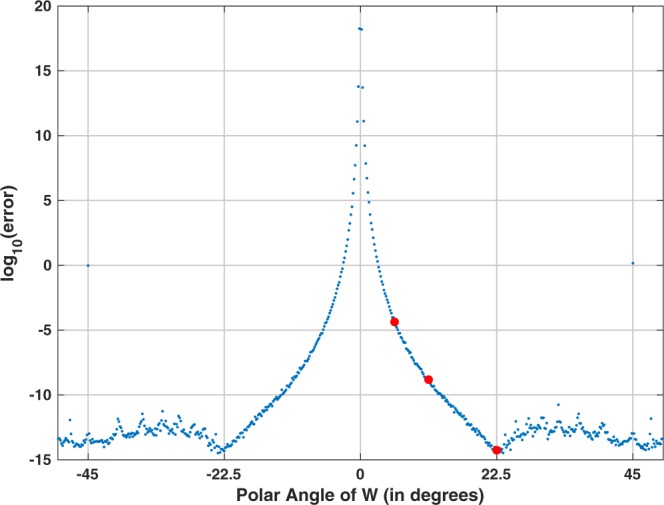
Absolute numerical error for 16-point chirp contours, shown as a function of the polar angle of *W*, discretized at 360°/2048. Each point represents the average error for ten random input vectors, each of unit length. The three red points indicate the errors for the three chirp contours shown in Fig. 1.

**Figure 3:**
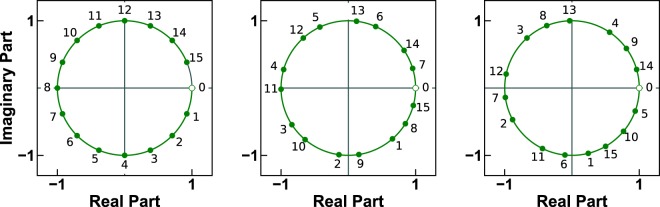
Three chirp contours with 16 points on the unit circle. The contour points are numbered in increasing order to illustrate the cases when a contour completes more than one revolution over the unit circle. From left to right, the polar angle of the parameter *W* is: 22.5°, 49.0°, and 76.0°.

  Figure 2 shows the numerical error for the sequential application of the CZT followed by the ICZT for a transform of size 16. Each point represents the average numerical error for 10 randomly generated unit-length complex input vectors. The numerical error is plotted as a function of the polar angle of the transform parameter *W*. The red points indicate the numerical error for the three contours from Fig. 1. For these contours, the error decreases as the samples cover larger fractions of the circle. The error for the rightmost contour is very small and is close to the machine epsilon. Increasing the angle of *W* above 22.5° eventually wraps the chirp contour over the unit circle more than once (see Fig. 3). The behavior of the error in those cases is more complicated and is related to Farey sequences.

## Related Work

There have been several unsuccessful attempts^11–14^ to derive an efficient inverse chirp z-transform (ICZT) algorithm. Most of these attempts have focused on the special case of inverting the CZT for chirp contours on the unit circle. In one case^13^, a modified version of the forward transform, in which the circular chirp contour was traversed in the opposite direction, was presented as the ICZT transform. This approach, however, does not really invert the forward CZT in the general case, i.e., for logarithmic spiral contours. This approach also doesn’t work for all chirp contours that lie on the unit circle, because the inverse of the Vandermonde matrix used by the forward transform is not, in general, a Vandermonde matrix. Traversing the contour in reverse order is equivalent to using another Vandermonde matrix as the transformation matrix. Our paper provides experimental results that show that this method does not work.

The literature describes two algorithms that generalize the FFT on the unit circle: the *Chirp Transform Algorithm* (CTA)^15^ and the *Fractional Fourier Transform* (FRFT)^16,17^. Both algorithms are special cases of the CZT for chirp contours that lie on the unit circle, but the FRFT contours always start at the complex point (1, 0). It has been estimated^18^ that the inverse chirp transform can be performed in 2M(*n*) + *O*(*n*), where M(*n*) can be taken in $O(n\ {\rm{\log }}(n)\ {\rm{\log }}({\rm{\log }}(n)))$.

As described in Supplementary Section S3, the CTA and the FRFT can each be implemented with a single call to the CZT algorithm. Supplementary Section S3 also describes how to implement the inverse CTA and the inverse FRFT algorithms, which have not been described in the literature until now. We named these algorithms ICTA and IFRFT. They are implemented as special cases of the ICZT algorithm. Both algorithms run in $O(n\,{\rm{\log }}\,n)$ time and use *O*(*n*) memory.

The FFT and IFFT are two very similar algorithms. They are also very stable numerically. The reason for this is that the harmonically-spaced frequency components that they use are orthogonal. This condition doesn’t hold for the CZT and the ICZT, which explains why the ICZT algorithm is substantially different from the CZT algorithm. The numerical accuracy of the ICZT depends on the values of its parameters *A* and *W*.

A different, but related, problem is generalizing the FFT or the IFFT to nonequispaced sampling points on the unit circle^19^. This problem is solved with approximate or iterative algorithms^19–22^. The accuracy and speed of approximate algorithms depend on the desired precision^20^, which is often controlled by an oversampling parameter^22^. The computational complexity of iterative algorithms^19,22^ depends on the condition number of the problem, i.e., they may require many iterations to converge. In contrast, the CZT and ICZT algorithms are exact and their computational complexity depends only on the problem size.

## Summary of the CZT and ICZT Algorithms

### Forward CZT

The chirp z-transform (CZT) is defined^7^ as follows:

1 $${{\rm{X}}}_{k}=\mathop{\sum }\limits_{j=0}^{N-1}{{\rm{x}}}_{j}\ {A}^{-j}\ {W}^{jk},\quad k=0,1,\ldots ,M-1,$$

where **x** is the input vector of length *N* and **X** is the output vector of length *M*. Using matrix notation this can be stated as:

2 $${\bf{X}}={\boldsymbol{W}}{\bf{A}}\ {\bf{x}}.$$

In this formula, **A** is the following diagonal matrix

3 $${\bf{A}}={\rm{diag}}({A}^{-0},{A}^{-1},\ldots ,{A}^{-(N-1)})$$

and ***W*** is the following Vandermonde matrix:

4 $${\boldsymbol{W}}=\left[\begin{array}{cccc}{W}^{0\cdot 0} & {W}^{1\cdot 0} & \ldots  & {W}^{(N-1)\cdot 0}\\ {W}^{0\cdot 1} & {W}^{1\cdot 1} & \ldots  & {W}^{(N-1)\cdot 1}\\ \vdots  & \vdots  & \ddots  & \vdots \\ {W}^{0\cdot (M-1)} & {W}^{1\cdot (M-1)} & \ldots  & {W}^{(N-1)\cdot (M-1)}\end{array}\right].$$

Using Bluestein’s substitution^9^, i.e., $jk=(\,{j}^{2}+{k}^{2}-{(k-j)}^{2})/2$, the matrix ***W*** can be expressed as ${\boldsymbol{W}}={\bf{P}}\widehat{{\boldsymbol{W}}}{\bf{Q}}$. That is, it is equal to a product of three matrices where $\widehat{{\boldsymbol{W}}}$ is the following Toeplitz matrix

5 $$\widehat{{\boldsymbol{W}}}=\left[\begin{array}{llll}{W}^{-\frac{{(0-0)}^{2}}{2}} & {W}^{-\frac{{(0-1)}^{2}}{2}} & \cdots \  & {W}^{-\frac{{(0-(N-1))}^{2}}{2}}\\ {W}^{-\frac{{(1-0)}^{2}}{2}} & {W}^{-\frac{{(1-1)}^{2}}{2}} & \cdots \  & {W}^{-\frac{{(1-(N-1))}^{2}}{2}}\\ \vdots  & \vdots  & \ddots  & \vdots \\ {W}^{-\frac{{((M-1)-0)}^{2}}{2}} & {W}^{-\frac{{((M-1)-1)}^{2}}{2}} & \cdots \  & {W}^{-\frac{{((M-1)-(N-1))}^{2}}{2}}\end{array}\right]$$

and **P** and **Q** are the following two diagonal matrices:

6 $${\bf{P}}={\rm{diag}}\left({W}^{\frac{{0}^{2}}{2}},{W}^{\frac{{1}^{2}}{2}},\cdots \ ,{W}^{\frac{{(M-1)}^{2}}{2}}\right)\quad \,{\rm{and}}\,\quad {\bf{Q}}={\rm{diag}}\left({W}^{\frac{{0}^{2}}{2}},{W}^{\frac{{1}^{2}}{2}},\cdots \ ,{W}^{\frac{{(N-1)}^{2}}{2}}\right).$$

Thus, the CZT algorithm can be viewed as an efficient implementation of the following matrix equation:

7 $${\bf{X}}={\bf{P}}\ (\widehat{{\boldsymbol{W}}}({\bf{Q}}\left({\bf{A}}\ {\bf{x}}\right))).$$

By exploiting the structure of the matrices, the output vector **X** can be computed in *O*(*n* log *n*) time, where $n=\max (M,N)$.

Algorithm S5 in Supplementary Section S2 gives the pseudo-code for the forward CZT.

### Inverse CZT

In the square case, i.e., when *M* = *N*, the ICZT can be stated^10^ by inverting Eq. (7), which leads to:

8 $${\bf{x}}={{\bf{A}}}^{-1}{{\bf{Q}}}^{-1}\ {{\boldsymbol{{\hat{W}}}}}^{-1}\ {{\bf{P}}}^{-1}\ {\bf{X}}.$$

Because **A**, **Q**, and **P** are diagonal matrices, it is straightforward to compute the inverse matrices **A**^−1^, **Q**^−1^, and **P**^−1^. The symmetric Toeplitz matrix $\widehat{{\boldsymbol{W}}}$ can be inverted^10^ using a special case of the Gohberg–Semencul formula^23,24^. In other words, the inverse matrix ${{\boldsymbol{{\hat{W}}}}}^{-1}$ is given by:

9 $${{\boldsymbol{{\hat{W}}}}}^{-1}=\frac{1}{{{\rm{u}}}_{0}}(\boldsymbol{\mathcal{A}}\ {\boldsymbol{\mathcal{A}}}^{T}-{\boldsymbol{\mathcal{D}}}^{T}\boldsymbol{\mathcal{D}}),$$

where $\boldsymbol{\mathcal{A}}$ is a lower-triangular Toeplitz matrix and $\boldsymbol{\mathcal{D}}$ is an upper-triangular Toeplitz matrix. Both $\boldsymbol{\mathcal{A}}$ and $\boldsymbol{\mathcal{D}}$ are defined by the same generating vector **u** = (u_0_, u_1_, u_2_, …, u_*n*−1_), i.e.,

10 $$\boldsymbol{\mathcal{A}}=\left[\begin{array}{ccccc}{{\rm{u}}}_{0} & 0 & 0 & \cdots \  & 0\\ {{\rm{u}}}_{1} & {{\rm{u}}}_{0} & 0 & \cdots \  & 0\\ {{\rm{u}}}_{2} & {{\rm{u}}}_{1} & {{\rm{u}}}_{0} & \cdots \  & 0\\ \vdots  & \vdots  & \vdots  & \ddots  & \vdots \\ {{\rm{u}}}_{n-1} & {{\rm{u}}}_{n-2} & {{\rm{u}}}_{n-3} & \cdots \  & {{\rm{u}}}_{0}\end{array}\right],\quad \boldsymbol{\mathcal{D}}=\left[\begin{array}{ccccc}0 & {{\rm{u}}}_{n-1} & {{\rm{u}}}_{n-2} & \cdots \  & {{\rm{u}}}_{1}\\ 0 & 0 & {{\rm{u}}}_{n-1} & \cdots \  & {{\rm{u}}}_{2}\\ 0 & 0 & 0 & \cdots \  & {{\rm{u}}}_{3}\\ \vdots  & \vdots  & \vdots  & \ddots  & \vdots \\ 0 & 0 & 0 & \cdots \  & 0\end{array}\right].$$

The value of u_*k*_ for each *k* ∈ {0, 1, …, *n* − 1} is given by:

11 $${{\rm{u}}}_{k}={(-1)}^{k}\frac{{W}^{\frac{2{k}^{2}-(2n-1)k+n(n-1)}{2}}}{\mathop{\prod }\limits_{s=1}^{n-k-1}({W}^{s}-1)\mathop{\prod }\limits_{s=1}^{k}({W}^{s}-1)}.$$

Combining these results leads to the following closed-form solution for the ICZT:

12 $${\bf{x}}=\frac{1}{{{\rm{u}}}_{0}}{{\bf{A}}}^{-1}{{\bf{Q}}}^{-1}\ (\boldsymbol{\mathcal{A}}\ {\boldsymbol{\mathcal{A}}}^{T}-{\boldsymbol{\mathcal{D}}}^{T}\boldsymbol{\mathcal{D}})\ {{\bf{P}}}^{-1}\ {\bf{X}}.$$

Algorithm 1 computes this expression in *O*(*n* log *n*) time by exploiting the structure of the matrices. See reference^10^ for more details, proofs, and the pseudo-code for ToeplitzMultiplyE and all of its dependencies.

**Algorithm 1:**
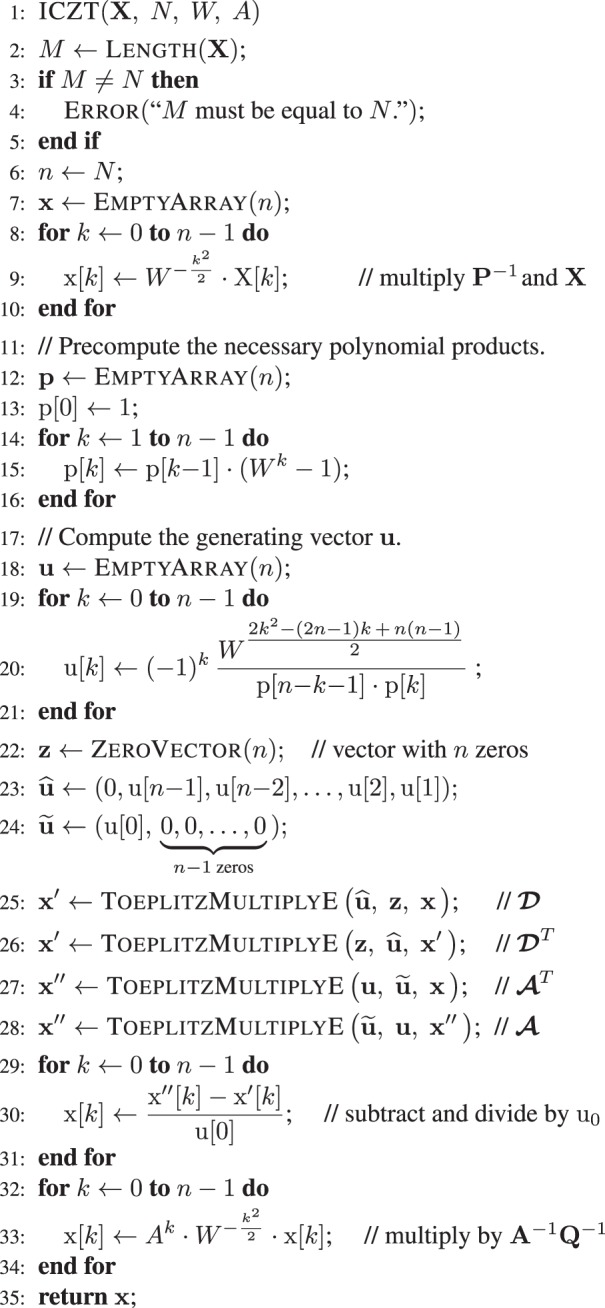
ICZT algorithm. Runs in *O*(*n* log *n*) time.

## Farey sequences and ICZT singularities

This section proves that the singularities of the ICZT on the unit circle are related to the elements of the Farey sequence of order *n* − 1, where *n* is the size of the transform.

**Definition 1**.  *A Farey sequence of order* *n* *is denoted by*  *F*_*n*_. *It consists of all rational numbers in the interval* [0, 1] *with an irreducible fraction representation*  *p*/*q*  *that satisfies the inequality*  *q* ≤ *n*,  *where n is a positive integer*.

A fraction *p*/*q* is irreducible if there is no other fraction *a*/*b* for which ∣*a*∣ < ∣*p*∣ or ∣*b*∣ < ∣*q*∣ such that *p*/*q* = *a*/*b*. For example, 0/1 is irreducible, but 0/5 is not. To give another example, both 2 ∕4 and 3 ∕6 are reducible to 1/2.

By mathematical convention, the numbers in each Farey sequence are sorted in increasing order. For example, the first five Farey sequences are equal to:

$$\begin{array}{lll}{F}_{1} & = & \left(\frac{0}{1},\ \frac{1}{1}\right),\\ {F}_{2} & = & \left(\frac{0}{1},\ \frac{1}{2},\ \frac{1}{1}\right),\\ {F}_{3} & = & \left(\frac{0}{1},\ \frac{1}{3},\ \frac{1}{2},\ \frac{2}{3},\ \frac{1}{1}\right),\\ {F}_{4} & = & \left(\frac{0}{1},\ \frac{1}{4},\ \frac{1}{3},\ \frac{1}{2},\ \frac{2}{3},\ \frac{3}{4},\ \frac{1}{1}\right),\\ {F}_{5} & = & \left(\frac{0}{1},\ \frac{1}{5},\ \frac{1}{4},\ \frac{1}{3},\ \frac{2}{5},\ \frac{1}{2},\ \frac{3}{5},\ \frac{2}{3},\ \frac{3}{4},\ \frac{4}{5},\ \frac{1}{1}\right).\end{array}$$

The Farey sequence of order *n* contains all elements of the Farey sequence of order *n* − 1 and some new elements that are unique to *F*_*n*_. Because *F*_*n*_ includes all elements of *F*_*n*−1_ and *F*_*n*−1_ includes all elements of *F*_*n*−2_, it follows that *F*_*n*_ includes all elements of *F*_*n*−2_ as well. In fact, *F*_*n*_ includes all elements of *F*_1_, *F*_2_, …, *F*_*n*−1_. This property becomes more clear if the Farey sequences shown above are rewritten with extra space between some of the terms as shown below:

$$\begin{array}{rcl}{F}_{1} & = & (\begin{array}{ccccccccccc}\frac{0}{1}, & \, & \, & \, & \, & \, & \, & \, & \, & \hspace{4.5pc} & \frac{1}{1}\end{array}),\\ {F}_{2} & = & (\begin{array}{ccccccccccc}\frac{0}{1}, & \, & \, & \, & \hspace{2.2pc} & \frac{1}{2}, & \, & \, & \, & \hspace{2pc} & \frac{1}{1}\end{array}),\\ {F}_{3} & = & (\begin{array}{ccccccccccc}\frac{0}{1}, & \, & \hspace{1.2pc} & \frac{1}{3}, & \hspace{.7pc} & \frac{1}{2}, & \hspace{.7pc} & \frac{2}{3}, & \, & \hspace{1pc} & \frac{1}{1}\end{array}),\\ {F}_{4} & = & (\begin{array}{ccccccccccc}\frac{0}{1}, & \hspace{.7pc} & \frac{1}{4}, & \frac{1}{3}, & \hspace{.7pc} & \frac{1}{2}, & \hspace{.7pc} & \frac{2}{3}, & \frac{3}{4}, & \, & \frac{1}{1}\end{array}),\\ {F}_{5} & = & (\begin{array}{ccccccccccc}\frac{0}{1}, & \frac{1}{5}, & \frac{1}{4}, & \frac{1}{3}, & \frac{2}{5}, & \frac{1}{2}, & \frac{3}{5}, & \frac{2}{3}, & \frac{3}{4}, & \frac{4}{5}, & \frac{1}{1}\end{array}).\end{array}$$

This visualization also illustrates another property of Farey sequences: the number of times that a fraction *p*/*q* ∈ *F*_*n*_ appears in the sequences *F*_1_, *F*_2_, …, *F*_*n*−1_ is equal to *n* − *q*. For example, the fraction 1/3 appears 5 − 3 = 2 times in *F*_1_, *F*_2_, *F*_3_, and *F*_4_.

The next definition links Farey fractions to polar angles.

**Definition 2**.  *Each angle*  *θ*  *that can be expressed in radians as*  *θ* = 2*πp*/*q*  *is a Farey angle of order n if p/q is an irreducible fraction that is an element of the Farey sequence of order n*.

Note that each Farey sequence includes two distinct elements 0/1 and 1/1. However, these two fractions map to the same Farey angle, i.e., 0 and 2*π* are equivalent.

The following theorem proves that for each Farey angle the corresponding complex exponential e^*i*2*πp*/*q*^ is a root of unity of order *q*. The converse is also true: each root of unity corresponds to a Farey angle and the order of the root of unity determines the order of the Farey sequence in which the fraction *p*/*q* first appears.

**Theorem 1**.  *Let*  $\theta \in \left[\left.0,2\pi \right)\right.$  *be an angle expressed in radians*. *Then*,  *θ*  *is a Farey angle of order n if and only if there is an integer*  *q* ∈ {1, 2, …, *n*} *such that the complex exponential*  e^*iθ*^  *is a root of unity of order*  *q*  *for some* *q* ≤ *n*.

*Proof*. (⇒) Suppose that *θ* is a Farey angle of order *n*. Then, *θ* = 2*πp*/*q*, where *p*/*q* ∈ *F*_*n*_. This implies that the complex exponential e^*iθ*^ is a root of unity of order *q*. That is,

13 $${({{\rm{e}}}^{i\theta })}^{q}={\left({{\rm{e}}}^{i2\pi \frac{p}{q}}\right)}^{q}={{\rm{e}}}^{i2\pi \frac{pq}{q}}={{\rm{e}}}^{i2\pi p}=1,$$

because *p* is an integer.

(⇐) Suppose that the complex exponential e^*iθ*^ is a root of unity of order *q* ≤ *n*, i.e.,

14 $${({{\rm{e}}}^{i\theta })}^{q}={{\rm{e}}}^{i\theta q}=1.$$

This equation implies that *θq* is an integer multiple of 2*π*. That is, without loss of generality, *θq* = 2*πp*, where *p* is a non-negative integer between 0 and *q* − 1. Therefore,

15 $$\theta =\frac{2\pi p}{q},$$

which implies that *θ* ∈ *F*_*q*_ ⊆ *F*_*n*_, as required.□

The next theorem ties the Farey angles that correspond to the elements of *F*_*n*−1_ to the singularities of Eq. (11), which defines the generating vector **u** that is used in the closed-form expression for the inverse chirp z-transform.

**Theorem 2**. *Let* $p/q\in {\mathbb{Q}}$ *be an irreducible fraction in the interval*  [0, 1]. *Let n be a positive integer and let*  $W={{\rm{e}}}^{i2\pi \frac{p}{q}}$. *If the fraction p/q is an element of the Farey sequence of order n  −  1, i.e*., *p*/*q* ∈ *F*_*n*−1_,  *then there is at least one element of the vector* **u** *for which the denominator in* Eq. (11) *is zero*.

*Conversely, if*  *p*/*q* ∉ *F*_*n*−1_, *then all elements of the vector*  **u**  *defined by* Eq. (11)  *have finite magnitudes and are well-defined*.

*Proof*. Because ∣*W*∣ = 1, the absolute value of the numerator in Eq. (11) is equal to 1 for each *k* ∈ {0, 1, 2, …, *n* − 1}.

(⇒) Suppose that *p*/*q* ∈ *F*_*n*−1_. Then, by definition, *q* ≤ *n* − 1. Therefore, there is at least one zero term in the product that defines the denominator of the element u_0_. More formally,

16 $$\begin{array}{ccccc}\mathop{\prod }\limits_{s=1}^{n-1}\ ({W}^{s}-1) & = & \mathop{\prod }\limits_{s=1,s\ne q}^{n-1}\ ({W}^{s}-1)\mathop{\prod }\limits_{s=q}^{q}\ ({W}^{s}-1) & = & \mathop{\prod }\limits_{s=1,s\ne q}^{n-1}({{\rm{e}}}^{i2\pi \frac{ps}{q}}-1)\mathop{\underbrace{({{\rm{e}}}^{i2\pi \frac{pq}{q}}-1)}}\limits_{0}=0.\end{array}$$

(⇐) Conversely, suppose that *p*/*q* ∉ *F*_*n*−1_. Then, *q* ≥ *n*. Because *p* and *q* are coprime, this implies that $ps/q\notin {\mathbb{Z}}$ for each *s* ∈ {1, 2, …, *n* − 1}. Therefore, each term in the denominator of each element u_*k*_ is non-zero.□

Supplementary Section S4 gives additional proofs and examples that perform the analysis using the rank of the Vandermonde matrix ***W*** in Eq. (4) instead of the elements of the generating vector **u**, which is defined by Eq. (11). In other words, the proof in this section is based on the singularities of the closed-form formula for the ICZT, which is implemented by the ICZT algorithm. The proof in Supplementary Section S4 is based on the rank of the ICZT transformation matrix. Because the two sets of singularities are the same, any algorithm that computes or approximates the ICZT on the unit circle will have singularities at Farey angles that correspond to the elements of *F*_*n*−1_, where *n* is the transform size. That is, the singularities at Farey angles are inherent to the mathematical problem; they are not introduced by Algorithm 1.

Supplementary Section S6 includes plots of the condition numbers for the transform matrix ***W*** for chirp contours with 16 and 32 points. The results show that the condition number also spikes near Farey angles. The shapes of the condition number plots are similar to the shapes of the numerical error plots. The condition numbers were computed from the singular value decomposition of the matrix ***W***. This was done using the following formula: ${\rm{cond}}\,({\boldsymbol{W}})={\sigma }_{\max }/{\sigma }_{\min }$, where ${\sigma }_{\max }$ is the largest singular value and ${\sigma }_{\min }$ is the smallest singular value of the matrix ***W***.

## Results

### Results for 16-point contours

This subsection summarizes the results of the first experiment, which studied the properties of the numerical error for circular chirp contours with 16 points. The number of points is too small for most practical applications, but the lessons learned from these contours allowed us to derive the error prediction formulas described later in the paper.

Figure 3 shows three different chirp contours that lie on the unit circle. Each contour has 16 points and each point is labeled with its index, a number between 0 and 15. The leftmost contour performs exactly one revolution. The other contours perform between 2 and 3 complete revolutions over the unit circle. In all three cases, the contours are traversed clockwise because the polar angle of *W* is positive. The reason for this is that chirp contours are defined in terms of the z-transform, which uses negative powers (see Supplementary Section S1).

**Figure 4:**
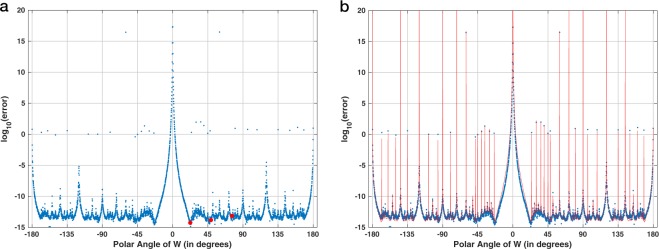
(**a**) Absolute numerical error of the CZT–ICZT procedure, as a function of the polar angle of *W* for 16-point chirp contours on the unit circle. The discretization step was 0.1°, i.e., there were 3600 angles. The three red points indicate the error for the three contours shown in Fig. 3. (**b**) The plot from (**a**) with the error prediction from Eq. (21) superimposed in red.

 Figure 4a shows the absolute numerical error of the CZT–ICZT procedure (see Methods) for 16-point contours on the unit circle. Fig. 4b shows the same plot, but with the numerical error predictions superimposed in red (see Eq. (21)). The horizontal axis corresponds to the polar angles of the parameter *W*, which are discretized at 0.1° intervals, i.e., there are 3600 angles. The vertical axis shows the decimal logarithm of the absolute numerical error. For each of the 3600 angles, the error was averaged over 10 randomly generated input vectors after computing the logarithm. The same random vectors were used to compute the error for each point. The three red points in the plot correspond to the three chirp contours shown in Fig. 3.

**Figure 5:**
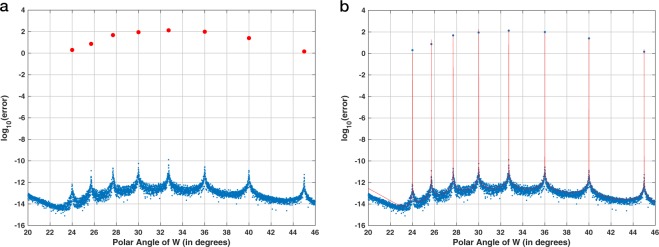
(**a**) Close-up of the harmonic hedgehog located between the first two red points in Fig. 4a. The numerical error spikes when the polar angle of *W* is close to an element of the set $\{\frac{36{0}^{\circ }}{15},\frac{36{0}^{\circ }}{14},\frac{36{0}^{\circ }}{13},\cdots \ ,\frac{36{0}^{\circ }}{8}\}$. The eight red points indicate the numerical error for these Farey angles. (**b**) The plot from (**a**) with the numerical error estimate superimposed in red.

 Figure 5a shows a close-up view of Fig. 4a for angles between 20° and 46°. The discretization step in this case is 0.005°, which is sufficient to reveal the finer structure of the error function. The figure shows that the numerical error spikes for angles that are close to the elements of the set $\{\frac{36{0}^{\circ }}{15},\frac{36{0}^{\circ }}{14},\frac{36{0}^{\circ }}{13},\cdots \ ,\frac{36{0}^{\circ }}{8}\}$. These angles form a subset of the harmonic sequence $(\frac{36{0}^{\circ }}{1},\frac{36{0}^{\circ }}{2},\frac{36{0}^{\circ }}{3},\cdots \ )$, which is why we called this pattern of spikes the *harmonic hedgehog*. These angles correspond to elements of the Farey sequence *F*_15_ between 1 ∕ 15 and 1 ∕ 8. The eight red points indicate the numerical errors for these Farey angles. The red points were explicitly added to the figure using Algorithm 3, i.e., their corresponding Farey angles were added to the list of discretized polar angles of *W* because even the finer discretization missed them. Figure 5b shows the same plot as in Fig. 5a, but with the error predictions superimposed in red. The singularities of the transform coincide with the discontinuities of the error prediction function. This figure also shows that the spread of the points in the empirical error plot is due to numerical rounding and the residual randomness that is not fully mitigated by averaging over only 10 input vectors.

The absolute numerical error for the Farey angles shown in Fig. 5a is large, but finite. The reason why the error is bounded and not infinite as predicted by the theoretical arguments in Theorem 2 is that, in practice, the IEEE-754 floating point numbers^25^ approximate these angles and their complex exponentials. Because *π* is irrational, the computational representation of all Farey angles except 0° is not exact, but approximate when they are expressed in radians. The floating-point computations use numbers that are very close, but still a tiny bit different from these Farey angles. This often leads to large, but finite, numerical errors.

### Results for 32-point contours

Supplementary Section S5 shows the results for 32-point chirp contours. They are similar to the results for the 16-point chirp contours, but the error function appears to be compressed horizontally by a factor of two. The harmonic hedgehog can also be observed, but it is now squeezed in the interval [11°, 23°] instead of the interval [22°, 46°] and has twice as many spikes. In this case, the ICZT singularities correspond to the Farey sequence *F*_31_ instead of *F*_15_. Supplementary Section S6 also includes plots of the condition number of the transform matrix ***W*** for 32-point contours.

### Results for 1024-point contours

The next set of experiments investigated the behavior of the numerical error of the CZT–ICZT procedure for contours with 1024 points on the unit circle. The results show that even for large transform sizes there are many values of *W* for which the ICZT can be computed accurately. These experiments also studied how the plot of the error function is affected by the discretization step for the polar angles of *W*.

**Figure 6:**
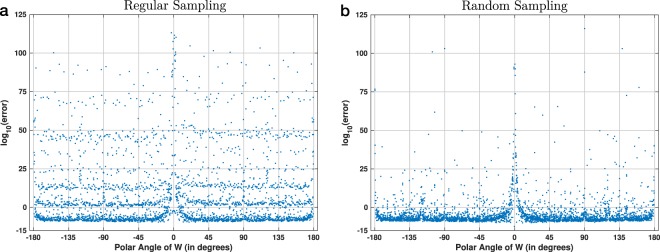
Absolute numerical error of the CZT–ICZT procedure for chirp contours with 1024 points on the unit circle. The results in (**a**) are for 3600 regularly-spaced angles with a discretization step of 0.1°. This discretization hits many Farey angles of lower orders, which leads to the layering of the numerical error function. See also Supplementary Fig. S3. The results in (**b**) are for 3600 polar angles that were randomly sampled from a uniform distribution. The random sampling is less likely to hit Farey angles, which explains the absence of the layering effect seen in (**a**).

Figure 6a shows the absolute numerical error for 3600 polar angles of the transform parameter *W*. These angles were selected using a regularly-spaced sampling grid with a step of 0.1°. For each angle, the numerical error was averaged over 10 random input vectors (see Methods). This discretization hits many Farey angles of different orders, which leads to the stratified appearance of the plot. This layering effect is explained by the link between Farey sequences and the prime factorization of the number of regularly-discretized angles. See Supplementary Section S4 for more details.

Figure 6b shows another plot with 3600 points, also for a transform of size 1024. In this case, however, the angles were sampled at random from a uniform distribution. The resulting pattern appears to be closer to the overall shape of the error function from the previous subsections, in which the transform size was smaller. The reason for the shape difference between Fig. 6a and Fig. 6b is that the random sampling is unlikely to select a Farey angle where the ICZT is singular.

**Figure 7:**
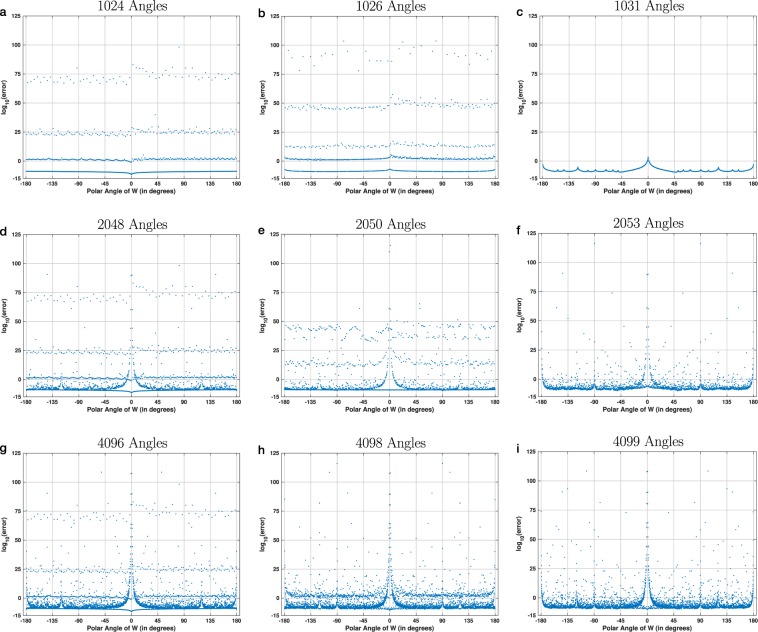
Absolute numerical error as a function of the polar angle of the transform parameter *W*, plotted for chirp contours with 1024 points on the unit circle. The nine plots illustrate the variety of shapes that the error function can take depending on the choice of discretization. In all plots, the size of the transform is fixed at 1024. What varies is the number of angles, i.e., polar angles of *W* that are discretized using regularly-spaced intervals. Each point in each plot shows the average value of the absolute error, computed with the CZT–ICZT procedure over 10 random input vectors. The top row, i.e., plots (**a**–**c**), shows the results for the case when the number of regularly-spaced polar angles is close to the number of points on the chirp contour, i.e., 1024. The second and the third row show the results when the number of angles is approximately 2 times and 4 times greater than the size of the transform, respectively. The left column, i.e., plots (**a**,**d**,**g**), shows the results for the case when the number of angles is a power of two. The plots in the middle column are for discretizations with 1026, 2050, and 4098 angles, which are composite numbers that are not powers of two. The right column shows plots for the case when the number of points is a prime number.

Figure 7 further explores the relationship between the error function and the discretization step. It shows nine plots of the absolute numerical error, computed for 1024-point chirp contours. These plots have completely different shapes, even though they were all computed for a transform of size 1024. In most cases, changing the number of regularly-sampled angles even a little bit leads to a different shape of the error function. Interestingly, the error function appears stratified in all six plots in the first two columns of Fig. 7, with different numbers and locations of the layers. This layering is absent in the last column of the figure, because these plots are drawn for a prime number of discretized polar angles. In all three cases, the prime number is greater than the transform size, which prevents hitting any ICZT singularities exactly.

The bottom row of Fig. 7 shows three plots that were obtained by regular sampling of the polar angles of *W* when the number of samples is about 4 times greater than the number of contour points. More specifically, the plots were drawn with 4096, 4098, and 4099 samples, respectively. Both 4096 and 4098 share integer factors with 1024, which explains the presence of layers in Fig. 7g,h. There are more layers in Fig. 7g compared to Fig. 7h because 1024 shares more factors with 4096 than 4098. There is no layering in Fig. 7i because 4099 and 1024 are coprime. Nevertheless, for some angles in Fig. 7i, the numerical error is high because these angles are sufficiently close to a nearby Farey angle of order less than 1024.

In other words, a plot in which the horizontal axis is discretized using a fixed step can lead to a misleading picture of the error. The behavior of the error may be completely different between the discretized points. The shape of the plot depends both on the sampling procedure and on the distribution of Farey angles. As shown below, the number of Farey angles where the transform is singular grows quadratically with the size of the transform, which makes it difficult to draw a complete plot of the error function for large values of *n*.

As proven in Theorem 2, the singularities of the ICZT of size *n* on the unit circle are related to the elements of the Farey sequence of order *n* − 1. Table 1 shows that as *n* increases the length of the Farey sequence *F*_*n*−1_ also increases^26^. These values imply that the number of singularities grows faster than the transform size. This growth is roughly quadratic, which follows from the popular approximation formula $| {F}_{n}|  \sim \frac{3{n}^{2}}{{\pi }^{2}}$ for the length of *F*_*n*_ (see ref. ^27^, p. 268 and ref. ^28^, p. 156).

**Table 1:** Length of the Farey sequence *F*_*n*−1_ as a function of *n*. The ICZT of size *n* has ∣*F*_*n*−1_∣ singularities when ∣*W*∣ = 1.

*n*	16	32	64	128	256	512	1024	2048
∣*F*_*n*−1_∣	73	309	1,229	4,959	19,821	79,597	318,453	1,274,563

The large number of singularities makes it more difficult to draw and interpret the error plots. For example, when *N* = 1024 there are 318453 singularities. In other words, the number of singularities is approximately two orders of magnitude larger than the number of all points plotted in Fig. 6a. This explains why even a small change in the discretization of the angles may lead to a completely different numerical error profile, as illustrated in Fig. 7.

Some discretizations select more Farey angles of small orders than others. For example, the discretization used in Fig. 6a hits many of these singularities and leads to the layering of the error function (see also Fig. S3, which colors each point based on its corresponding Farey order). The discretizations with prime number of angles shown in Fig. 7c,f,i don’t hit any small-order Farey angles exactly. This is true for any regular discretization in which the prime number is greater than or equal to the transform size. The next two subsections use discretizations with 4099 angles, which is a large prime number.

### Results for 2048-point contours

The next experiment studied the behavior of the error function for contours with 2048 points. Our previous experience with the ICZT indicated that the numerical accuracy decreases for contours with large number of points^10^. This is consistent with previous observations that Vandermonde systems can be ill-conditioned and should be solved with double precision or higher^29^. Interestingly, it was also shown that some ill-conditioned Vandermonde systems can be solved with high precision^30–32^. In our previous work, however, the chirp contours were off the unit circle, i.e., they were expanding or contracting logarithmic spiral contours. We wanted to check if additional numerical precision is still needed for contours with thousands of points that are restricted to lie on the unit circle.

**Figure 8:**
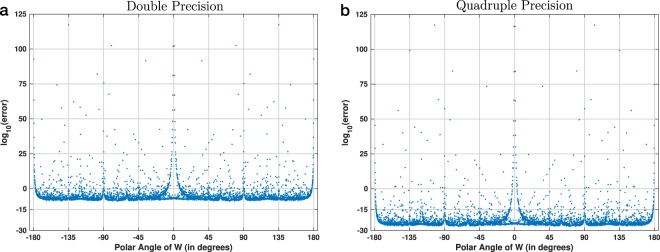
Absolute numerical error for the CZT–ICZT procedure, computed with two different floating-point precisions. In both plots, the results are for 2048-point contours on the unit circle and for 4099 polar angles of *W* that were discretized using regular intervals. The results in (**a**) were computed with double-precision, which is implemented natively in modern CPUs (i.e., 64-bit numbers in IEEE-754 format)^25^. The results in (**b**) were computed with 128-bit floating-point numbers, i.e., quadruple precision, implemented by GCC’s *libquadmath* library^33^ through an interface in *Boost Multiprecision*^34^.

Figure 8 shows the absolute numerical error of the CZT–ICZT procedure for 2048-point contours. These results are for 4099 polar angles that were discretized using regular intervals. The two plots were computed with two different floating-point precisions. Figure 8a shows the results for double precision, which is implemented natively in modern CPUs. Figure 8b shows a different plot that was computed with quadruple precision. It was generated using the *libquadmath* library^33^ through an interface provided by *Boost Multiprecision*^34^, which makes it easier to use various floating point formats in C++. The plots show that the numerical accuracy can be boosted by increasing the precision of the floating-point numbers. Switching from double precision to quadruple precision reduces the error by approximately 16 orders of magnitude. The overall shape, however, remains mostly the same.

The results also indicate that the transforms are computable for *N* = 2048 using only double precision. Unless the polar angle of *W* is close to a Farey angle, the error is usually small when the chirp contour is on the unit circle. Nevertheless, if additional numerical accuracy is needed, then one can switch from double to quadruple floating-point precision, which is becoming more popular and easier to use.

### Formulas for predicting the numerical error

This subsection states formulas that approximate the absolute numerical error for the sequential applications of the forward and inverse transforms, i.e., CZT followed by ICZT and vice versa. The formulas are given for the square case in which *M* = *N* and for chirp contours that lie on the unit circle. The error formulas for a more general case with logarithmic spiral contours that span a 360° arc off the unit circle are given in ref. ^10^.

The numerical error can be modeled using five terms: *U*_1_, *U*_2_, *U*_3_, *T*, and *B*. The first three terms are derived from the elements of the generating vector **u** (see Eq. (11)). For contours on the unit circle they are equal to:

17 $${U}_{1}={\rm{\log }}\,\sqrt{\mathop{\sum }\limits_{k=1}^{N-1}| {{\rm{u}}}_{k}{| }^{2}}=\frac{1}{2}{\rm{\log }}\,\mathop{\sum }\limits_{k=1}^{N-1}| {{\rm{u}}}_{k}{| }^{2},\quad {U}_{2}={\rm{\log }}\,\sqrt{\mathop{\sum }\limits_{k=0}^{N-1}| {{\rm{u}}}_{k}{| }^{2}}=\frac{1}{2}{\rm{\log }}\,\mathop{\sum }\limits_{k=0}^{N-1}| {{\rm{u}}}_{k}{| }^{2},\quad {U}_{3}=-{\rm{\log }}\,| {{\rm{u}}}_{0}| .$$

The term *U*_1_ is equal to the logarithm of the Euclidean norm of the first row of the matrix $\boldsymbol{\mathcal{D}}$, which includes all elements of **u** except u_0_  (see Eq. (10)). The term *U*_2_ is equal to the logarithm of the Euclidean norm of the first column of the matrix $\boldsymbol{\mathcal{A}}$, which is defined by Eq. (10). It is similar to *U*_1_, but also includes u_0_ in addition to all other elements. Finally, the term *U*_3_ models the division by u_0_ in Eq. (12). Thus, *U*_3_ is equal to the logarithm of $\frac{1}{| {{\rm{u}}}_{0}| }$, where u_0_ is not equal to zero by definition. For the error formulas to be accurate, these terms must be computed using the same numerical precision that is used by the ICZT algorithm, preferably using the same version of the vector **u**.

The fourth term, *T*, is the sum of three simpler terms^10^. In this special case, however, they are all equal to $\frac{1}{2}{\rm{\log }}\,N$ because the transform parameters *A* and *W* have a magnitude of 1, i.e., ∣*A*∣ = ∣*W*∣ = 1. Thus, the term *T* is equal to:

18 $$T=\frac{3}{2}{\rm{\log }}\,N.$$

The formulas also include an offset term, *B*, that depends on the size of the transform, *N*, and the number of precision bits, *p*, used in the calculations. This offset determines the base level of the error function. It is defined as follows:

19 $$B=-p\,{\rm{\log }}\,2+{C}_{1}\ {\rm{\log }}\,N+{C}_{2},$$

where *C*_1_ and *C*_2_ are implementation-dependent constants. For 64-bit and 128-bit IEEE-754 floating-point numbers^25^, the value of *p* is set to 53 and 113, respectively.

#### Error formula for the CZT followed by the ICZT

The absolute numerical error for a sequential application of the CZT followed by the ICZT is equal to the Euclidean distance between the original input vector **x** and the computed vector $\widehat{{\bf{x}}}$. That is,

20 $$E=\parallel \widehat{{\bf{x}}}-{\bf{x}}\parallel .$$

The error values for the CZT–ICZT procedure can be analytically approximated using the following formula:

21 $${\rm{\log }}\,E\approx {U}_{1}+{U}_{2}+{U}_{3}+T+B+{\rm{\log }}\,\parallel {\bf{x}}\parallel .$$

#### Error formula for the ICZT followed by the CZT

The absolute error of the ICZT–CZT procedure is

22 $$E=\parallel \widehat{{\bf{X}}}-{\bf{X}}\parallel ,$$

where **X** is the true output vector and $\widehat{{\bf{X}}}$ is the computed output vector. The log of the error is approximately equal to:

23 $${\rm{\log }}\,E\approx {U}_{1}+{U}_{2}+{U}_{3}+T+B+{\rm{\log }}\,\parallel {\bf{X}}\parallel .$$

#### Evaluation of the Error Prediction Formulas

The accuracies of Eqs. (21) and (23) were evaluated using the two procedures described above. The difference between predicted and empirically observed errors was quantified using the *R*^2^ coefficient (see Methods). Table 2 shows the means and the standard deviations of the *R*^2^ coefficients for the two experimental procedures and for transform sizes between 16 and 2048. In all cases, the results were computed by averaging the *R*^2^ values from 10 independent runs of the corresponding procedure, where each run used 10 random input vectors. For each value of *N*, the fits used 4099 regularly-discretized polar angles of *W*. The average *R*^2^ value increases with the size of the transform and approaches 1, which indicates that the formulas predict the numerical error very well. The results also show that there is no substantial difference between the *R*^2^ values for the two experimental procedures.

**Table 2:** *R*^2^ fits for the predicted numerical error for regularly-sampled polar angles of the transform parameter *W*.

*N*	CZT–ICZT		ICZT–CZT	
	Avg.	Std.	Avg.	Std.
16	0.96977	3.43535 × 10^−4^	0.97642	2.94905 × 10^−4^
32	0.98703	8.90973 × 10^−5^	0.98932	1.15295 × 10^−4^
64	0.99453	3.43848 × 10^−5^	0.99520	1.75565 × 10^−5^
128	0.99656	7.65721 × 10^−6^	0.99680	1.40965 × 10^−5^
256	0.99752	5.36489 × 10^−6^	0.99758	4.72754 × 10^−6^
512	0.99823	3.00392 × 10^−6^	0.99824	2.84512 × 10^−6^
1024	0.99863	9.77751 × 10^−7^	0.99863	1.89758 × 10^−6^
2048	0.99871	8.76893 × 10^−7^	0.99871	1.55039 × 10^−6^

Additional results for the accuracy of the error prediction formulas are given in Supplementary Sections S7 and S8.

## Debunking the Reverse-as-Inverse Approach for Inverting the CZT on the Unit Circle

This section shows that a naïve but surprisingly popular approach for inverting the CZT by reversing the direction of the chirp contour is incorrect. The essence of this reverse-as-inverse approach is captured by a formula from ref. ^13^ that is reproduced below:

24 $${\rm{ICZT}}(X(k))={[{\rm{CZT}}(X{(k)}^{\ast })]}^{\ast }.$$

The formula attempts to express the ICZT of the vector *X*(*k*) by conjugating all elements of the CZT of the vector *X*(*k*)^*^, where ^*^ denotes elementwise conjugation. In matrix form, Eq. (24) can be stated as follows:

25 $${\bf{y}}=\overline{{\boldsymbol{W}}{\bf{A}}\ \bar{{\bf{X}}}}=\bar{{\boldsymbol{W}}}\ \bar{{\bf{A}}}\ {\bf{X}}.$$

In this formula, **y** is the resulting vector, $\bar{{\boldsymbol{W}}}$ is the matrix obtained by conjugating all elements of the Vandermonde matrix ***W*** from Eq. (4), $\bar{{\bf{A}}}$ is the matrix obtained by conjugating all elements of the diagonal matrix **A** from Eq. (3), and **X** is the ICZT input vector. Instead of conjugating all elements of the two matrices, it is possible to compute the same result using the conjugates of the transform parameters *A* and *W*. That is, the vector **y** in Eq. (25) can be expressed as:

26 $${\bf{y}}={\rm{CZT}}({\bf{X}},M,\bar{W},\bar{A}).$$

If the chirp contour lies on the unit circle, then this formula reflects its starting point with respect to the real axis and also reverses its winding direction.

Ref. ^13^ also states that Eq. (24) needs to be interpreted “to within a scaling factor” that was not specified. That is, the equal sign denotes proportionality instead of equality. For unit-length input vectors **x** in the CZT–ICZT procedure (see Methods), optimal scaling for this approach can be achieved by normalizing the vector **y**, i.e.,

27 $$\widehat{{\bf{x}}}=\frac{{\bf{y}}}{\parallel {\bf{y}}\parallel }.$$

This ensures that $\parallel {\bf{x}}\parallel =\parallel \widehat{{\bf{x}}}\parallel =1$, i.e., the norm of the input vector is always equal to the norm of the output vector. Algorithm 2 implements the reverse-as-inverse approach, i.e., Eq. (26) with the scaling factor from Eq. (27). For error plots on the log scale, the triangle inequality implies that ${{\rm{\log }}}_{10}\,\parallel {\bf{x}}-\widehat{{\bf{x}}}\parallel \le {{\rm{\log }}}_{10}2\approx 0.3$, i.e., the absolute numerical error for the CZT followed by Algorithm 2 can never exceed 0.3 on the log scale. Thus, this scaling is favorable to this algorithm. Unfortunately, the results show that the algorithm has a systematic error that cannot be corrected by scaling.

The rest of this section describes three experiments that compared the accuracy of Algorithm 1 to the accuracy of Algorithm 2. In all experiments, the value of *A* was equal to 1, the value of *M* was equal to *N*, and the polar angle of *W* was sampled using regular discretization as described in Methods. The accuracies were measured with the CZT–ICZT procedure, also described in Methods, but with Algorithm 2 replacing the ICZT implementation in the second condition.

**Figure 9:**
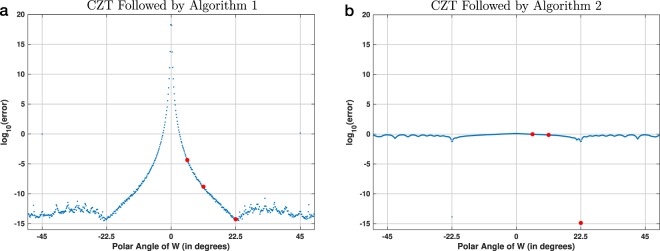
Absolute numerical error for 16-point chirp contours, shown as a function of the polar angle of *W* for two different experimental procedures: (**a**) the CZT followed by Algorithm 1, which implements the ICZT (this is the same as Fig. 2); and (**b**) the CZT followed by Algorithm 2, which uses the contour reversal approach proposed in reference^13^. The angles were discretized with a step of 360°/2048. The three red points in both (**a**) and (**b**) correspond to the chirp contours shown in Fig. 1. Each point in both plots represents the average error for 10 randomly generated unit-length input vectors.

 Figure 9 shows the results of the first experiment, which used circular chirp contours with 16 points. Figure 9a, which is a copy of Fig. 2, shows that Algorithm 1 accurately inverts the CZT for many polar angles of *W*. In contrast, Fig. 9b shows that the reverse-as-inverse approach is accurate only when this angle is equal to  − 22.5° or 22.5°. For all other angles the error is consistently and unacceptably high, i.e., the absolute numerical error (before the log) is close to 1, which is the norm of the input vector **x**. In both plots, the three red points correspond to the chirp contours shown in Fig. 1.

**Figure 10:**
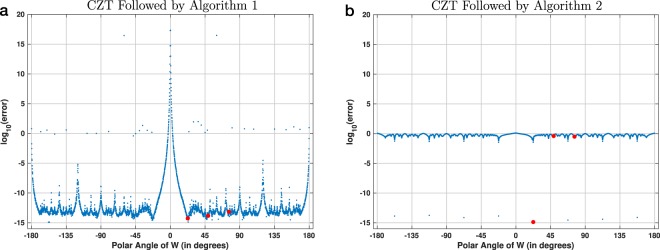
Visualization of the accuracy of two experimental procedures for 16-point chirp contours. This is similar to Fig. 9, but in this case the discretization step for the polar angle of *W* was set to 0.1°. The three red points in both plots correspond to the chirp contours shown in Fig. 3. The plot in (**a**) is the same as in Fig. 4a.

 Figure 10 shows the results of the second experiment, which also used 16-point circular chirp contours. In this case, the discretization used 3600 regularly-sampled polar angles. Figure 10a, which is a copy of Fig. 4a, shows the error using Algorithm 1. Figure 10b shows the error using the reverse-as-inverse approach. The three red points in each plot correspond to the three chirp contours shown in Fig. 3. Figure 10b shows that with Algorithm 2 the results are accurate only for the eight angles that correspond to the eight primitive roots of unity of order 16 (see also Supplementary Section S2).

**Figure 11:**
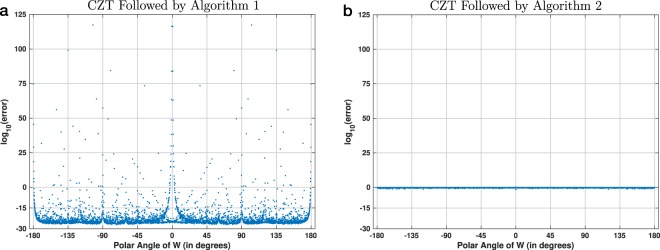
Visualization of the accuracy of two experimental procedures for 2048-point chirp contours. This is similar to Fig. 10, but in this case the discretization used 4099 regularly-spaced polar angles of *W* and the results were computed using quadruple precision (i.e., 128 bits) instead of double precision (i.e., 64 bits). The plot in (**a**) is the same as in Fig. 8b.

Finally, Fig. 11 shows the results of the third experiment, which used 2048-point circular chirp contours and 4099 regularly-discretized polar angles of *W*. In contrast to Figs. 9 and 10, which show results for double precision, the computations for this figure used quadruple precision. Figure 11a, which is a copy of Fig. 8b, shows the error using Algorithm 1. Figure 11b shows that the error using Algorithm 2 is again consistently large and proportional to ∣∣**x**∣∣. Because this discretization did not hit any primitive roots of unity of order 2048, there were no angles for which the reverse-as-inverse approach is accurate. The increased floating-point precision also did not help to reduce its error.

To summarize, the experiments showed that Algorithm 1 is accurate for most chirp contours and the numerical error of the CZT followed by the ICZT can be predicted using Eq. (21). In contrast, the reverse-as-inverse approach has large, systematic errors that are not affected by the numerical precision. It is accurate only when the chirp contour points coincide with the roots of unity of order *N*, i.e., only when the ICZT reduces to a permutation of the elements of the IFFT output vector. Algorithm 2 assumes that there is no interference between the frequency components, which is true only when they are orthogonal or, equivalently, when the value of *W* is a primitive root of unity of order *N*. For all other values of *W* the reverse-as-inverse approach fails to compute the ICZT.

## Discussion

This paper generalized the inverse fast Fourier transform (IFFT) to work with contours that perform partial or multiple revolutions on the unit circle. This was accomplished by analyzing the numerical error properties of the ICZT algorithm for a special case with circular chirp contours on the unit circle. The paper also proved that the ICZT singularities are tied to elements of Farey sequences, where the Farey order is smaller than the transform size. Formulas for predicting the numerical error were derived as well. The experiments showed that these formulas fit the empirically-observed numerical errors very well.

The FFT and the IFFT are restricted to use orthogonal, harmonically-spaced frequency components that are generated by the integer powers of the complex roots of unity. The CZT and the ICZT can use non-orthogonal frequency components, even for the special case with chirp contours on the unit circle. This additional flexibility, however, comes at a cost. For some values of the transform parameter *W* the ICZT could be very inaccurate. The error prediction formulas derived in this paper allow the practitioners to avoid these singularities. For most values of *W*, however, the ICZT is accurate and can be computed for large transform sizes.

The *Chirp Transform Algorithm* (CTA) and the *Fractional Fourier Transform* (FRFT) are two popular algorithms^15,16^ that can be viewed as special cases of the CZT for chirp contours on the unit circle. The corresponding inverse algorithms, however, have not been described until now. Supplementary Section S3 states two special cases of the ICZT algorithm that invert the CTA and the FRFT. We named these special cases the *Inverse Chirp Transform Algorithm* (ICTA) and the *Inverse Fractional Fourier Transform* (IFRFT).

This paper analyzed the numerical error of the ICZT algorithm for chirp contours on the unit circle that can perform partial or multiple revolutions. The numerical error for logarithmic spiral contours off the unit circle that span 360° was analyzed in our previous paper^10^. Future work could combine the insights from these two studies to derive error prediction formulas for logarithmic spiral contours off the unit circle that perform partial or multiple revolutions. In that case, the frequency components are not orthogonal and can also decay or grow exponentially. Future work could also try to derive bounds for the condition number of the transformation matrix and relate them to the error formulas derived in this paper.

## Methods

This section describes the methods that were used to evaluate the ICZT algorithm for chirp contours that lie on the unit circle. The experiments systematically varied the size of the transform and the sampling procedure for the polar angle of the parameter *W*. The transform parameter *A* was always set to 1 because changing its polar angle is equivalent to changing the polar angles of the elements of the ICZT output vector^10^, which does not affect the expected numerical error over all possible input vectors.

### CZT–ICZT procedure

The experiments measured the absolute numerical error for the sequential application of the CZT followed by the ICZT. The following five steps were repeated ten times for each value of the transform parameter *W*: (1) generate a complex input vector **x** by sampling its real and complex parts from a uniform distribution on the interval [−1, 1); (2) normalize the vector **x** so that its length is equal to 1; (3) use the vector **x** as input for the CZT algorithm, which results in the output vector $\widehat{{\bf{X}}}$; (4) use the ICZT algorithm with the vector $\widehat{{\bf{X}}}$ as input to compute the vector $\widehat{{\bf{x}}}$; and (5) compute the Euclidean distance between the vector $\widehat{{\bf{x}}}$ and the vector **x**. A different random seed was used for each repetition. The absolute numerical error of the CZT–ICZT procedure was set to the average Euclidean distance over these 10 repetitions. For experiments that varied the polar angle of *W*, the same 10 input vectors were used for all angles, i.e., the vectors were generated once and then re-used.

### ICZT–CZT procedure

Some of the experiments also used the ICZT–CZT procedure, in which the output of the ICZT algorithm was used as an input to the CZT algorithm. This procedure also consisted of five steps that were repeated ten times for each value of the transform parameter *W*: (1) generate a complex input vector **X** by sampling the real and complex parts of each of its elements from a uniform distribution on [−1, 1); (2) normalize the vector **X** so that it has unit length; (3) use the normalized vector **X** as input for the ICZT algorithm to compute the vector $\widehat{{\bf{x}}}$; (4) use the vector $\widehat{{\bf{x}}}$ as input for the CZT algorithm to compute the vector $\widehat{{\bf{X}}}$; and (5) compute the Euclidean distance between the vectors **X** and $\widehat{{\bf{X}}}$. A different random seed was used for each repetition. The absolute numerical error of this procedure was equal to the average Euclidean distance for these 10 repetitions. For experiments that varied the value of *W*, the input vectors were re-used as described above.

### Numerical error reporting

All numerical errors in this paper are reported on the log scale. For each polar angle of *W*, the Euclidean distance was averaged over 10 random input vectors after computing the logarithm. The experiments were designed such that the magnitude of the input vector and the magnitude of the expected output vector were both equal to 1. The results become exponentially more accurate as the error value decreases. For example, if the decimal logarithm of the absolute numerical error is −5, then the Euclidean distance between the result vector and the expected output vector is equal to 0.00001.

### Floating-point precisions

The experiments used two different floating-point precisions: (1) double-precision with 64 bits and (2) quadruple-precision with 128 bits. The double precision is implemented natively by modern CPUs. The quadruple precision was implemented using GCC’s *libquadmath*^33^. We used the interface from the *boost multiprecision* library^34^. In both cases the floating-point numbers were stored in IEEE-754 format^25^.

### Sampling of the polar angles of the transform parameter *W*

Two methods were used to sample the polar angles of *W*: (1) regularly-spaced sampling; and (2) random sampling from a uniform distribution. Both methods sampled *P* angles from the interval [0°, 360°). The figures, however, plot the angles between −180° and 180° because the largest peak of the error is centered at 0°, i.e., the interval [180°, 360°) is plotted as negative angles in the interval [−180°, 0°).

The regular sampling method used the following formula to select *P* polar angles *θ*_0_, *θ*_1_, *θ*_2_, …, *θ*_*P*−1_ to be evaluated:

28 $${\theta }_{k}=2\pi k/P,\quad \,{\rm{where}}\,k\in \{0,1,2,\ldots ,P-1\}\,{\rm{.}}\,$$

The random sampling method selected the polar angles by drawing i.i.d. samples from a uniform distribution on [0, 2*π*).

Given an angle *θ* in radians, the transform parameter *W* was computed using Euler’s formula: $W={{\rm{e}}}^{i\theta }=\cos \theta +i\ \sin \theta$.

### Reverse-as-Inverse algorithm

Algorithm 2 implements Eq. (24), which attempts to invert the CZT by reversing the direction of the chirp contour. As described in the paper, this approach does not really work.

**Algorithm 2:**
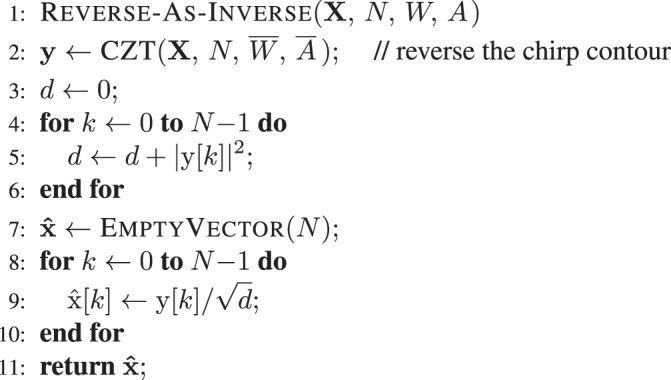
Reverse-as-Inverse algorithm with scaling.

### Enumerating Farey sequences

Farey sequences^28,35–37^ are related to the singularities of the ICZT when it is computed for circular chirp contours on the unit circle. In other words, there is a connection between addition of rational numbers and multiplication of complex numbers that lie on the unit circle. Each Farey sequence *F*_*n*_ is formed by all irreducible fractions *p*/*q* ∈ [0, 1], where *q* ≤ *n*. All Farey sequences share the *mediant* property. That is, if *a*/*b*, *p*/*q*, and *c*/*d* are any three consecutive elements of a Farey sequence, then *p*/*q* = (*a* + *c*)/(*b* + *d*).

The mediant property enables the enumeration of the elements of a Farey sequence by an algorithm^38^. Algorithm 3 shows the pseudo-code for an efficient procedure^38^ that generates a Farey sequence of order *n*. This procedure was used for some of the figures that explicitly included all ICZT singularities in a given subset of the parameter space.

**Algorithm 3:**
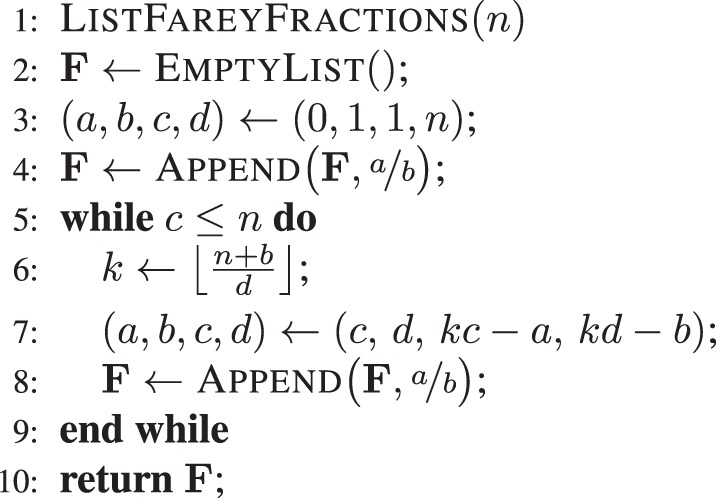
Generates the Farey sequence *F*_*n*_ in *O*(*n*^2^).

### Computing the *R*^2^ coefficient

The accuracy of the numerical error prediction formulas was evaluated using the *R*^2^ coefficient. That is, given two vectors **a** = (a_0_, a_1_, a_2_, …, a_*m*−1_) and **b** = (b_0_, b_1_, b_2_, …, b_*m*−1_), the *R*^2^ value was computed as follows:

29 $${R}^{2}=1-\alpha /\beta ,\quad \,{\rm{where}}\,\quad \alpha =\mathop{\sum }\limits_{k=0}^{m-1}{(\left({{\rm{a}}}_{k}-\bar{a}\right)-({{\rm{b}}}_{k}-\bar{b}))}^{2}\quad \,{\rm{and}}\,\quad \beta =\mathop{\sum }\limits_{k=0}^{m-1}{({{\rm{b}}}_{k}-\bar{b})}^{2}.$$

In this formula, $\bar{a}$ is the average of the elements in the vector **a** and $\bar{b}$ is the average of the elements in the vector **b**, i.e.,

30 $$\bar{a}=\left({{\rm{a}}}_{0}+{{\rm{a}}}_{1}+{{\rm{a}}}_{2}+\cdots +{{\rm{a}}}_{m-1}\right)/m\quad \,{\rm{and}}\,\quad \bar{b}=\left({{\rm{b}}}_{0}+{{\rm{b}}}_{1}+{{\rm{b}}}_{2}+\cdots +{{\rm{b}}}_{m-1}\right)/m.$$

In other words, the *R*^2^ coefficient is equal to 1 minus the fraction of the variance of the vector **b** that is unexplained by the vector **a**. Our approach centers the vectors (i.e., subtracts their averages) before computing *R*^2^. Thus, the constant offset $\bar{b}-\bar{a}$ does not affect the *R*^2^ value.

In the experiments, the vector **a** was set to the predicted logarithms of the numerical errors. The vector **b** was set to the logarithms of the numerical errors that were computed by either the CZT–ICZT or the ICZT–CZT procedure.

### Inversion of Toeplitz matrices

The CZT matrix is equal to the product of a Vandermonde matrix and a diagonal matrix. Bluestein’s substitution^9^ expresses the Vandermonde matrix as the product of two diagonal matrices and a Toeplitz matrix. Thus, the key to the ICZT algorithm is finding a computationally efficient way to invert this Toeplitz matrix.

The Gohberg–Semencul formula^23,24^ expresses the inverse of a Toeplitz matrix as a difference between two products of upper-triangular and lower-triangular Toeplitz matrices. These four matrices can be described by two generating vectors **u** and **v**, but the formula does not specify how to find these vectors. The matrix used by the CZT, however, is a special case of a symmetric Toeplitz matrix. For this matrix there is a special case of the Gohberg–Semencul formula with just one generating vector^10^. Furthermore, we were able to express the elements of this vector in terms of the transform parameter *W*. This led to an efficient ICZT algorithm that runs in *O*(*n* log *n*) time.

### Efficient Toeplitz–vector multiplication

The ICZT algorithm uses fast FFT-based subroutines for multiplying a Toeplitz matrix by a vector^10^. There are at least two different approaches for computing these products in *O*(*n* log *n*) time: (1) embedding the Toeplitz matrix into a larger circulant matrix (see ref. ^39^, p. 202) and (2) expressing the Toeplitz matrix as a sum of a circulant matrix and a skew-circulant matrix of the same shape using Pustylnikov’s decomposition^40,41^, see also ref. ^42^, p. 40 and ref. ^43^, p. 66.

Both subroutines multiply a circulant matrix or a skew-circulant matrix^44^ by a vector in *O*(*n* log *n*) time (i.e., fast circular convolution). The term *f*-circulant with *f* = −1 is also used in the literature to refer to skew-circulant matrices^43,45^. The required FFT sizes and the order of the operations performed by these two approaches are different. Their numerical accuracy, however, is similar. In all experiments described in this paper we used the embedding approach.

## Supplementary information

Supplementary Information.

## Data Availability

All data and procedures are described in the main paper or in the supplementary information.
