# Supplementary material for: Numerical error analysis of the ICZT algorithm for chirp contours on the unit circle
Source: Sci Rep. 2020 Mar 17;10:4852. doi: 10.1038/s41598-020-60878-7 (PMC7078310; doi:10.1038/s41598-020-60878-7)
Supplement: Supplementary file 1 — Supplementary Information. [file 41598_2020_60878_MOESM1_ESM.pdf]

Supplementary Information for "Numerical error analysis of the ICZT algorithm for chirp contours on the unit circle"

Vladimir Sukhoy[1] & Alexander Stoytchev[1,*]

[1] Department of Electrical and Computer Engineering, Iowa State University, Ames, IA 50011, USA. Correspondence and requests for materials should be addressed to A.S. (email: alexs@iastate.edu).

S1. Definitions for Contours and Transforms

By mathematical convention, positive angles correspond to counter-clockwise rotations and negative angles correspond to clockwise rotations (i.e., the right-hand rule). This convention, however, is violated for the winding direction of chirp contours. That is, *positive polar angles of W correspond to clockwise rotations*. The reason for this break with convention is that the CZT was defined with the z-transform, which uses negative powers, instead of the power series, which uses positive powers. The right-hand rule, however, still holds for the polar angle of the transform parameter A. This section clarifies these technical details using examples and explicit definitions.

A contour is a list of complex numbers that is derived from the transform parameters. Each complex number specifies a frequency component vector, the elements of which are equal to the integer powers of this number. For the DFT and the CZT, each element of the output vector is equal to the complex inner product between the input vector and the corresponding frequency component vector. The inverse transforms, i.e., the IDFT and the ICZT, map the output vector back to the input vector. A contour is shared by a matching pair of forward and inverse transforms, i.e., when they have the same parameters.

We will start by defining the Fourier contour that is used by the DFT and the IDFT (and, indirectly, by their fast implementations using the FFT and the IFFT algorithms).

Definition 3. ***Fourier contour.***
A Fourier contour consists of N points $\mathrm{c}_0, \mathrm{c}_1, \mathrm{c}_2, \ldots, \mathrm{c}_{N-1}$ in the complex plane, arranged in a counter-clockwise direction on the unit circle starting from the point $(1, 0)$. The coordinates of the k-th point, c_k, are given by the following formula:

$$\mathrm{c}_k = \mathrm{e}^{\frac{i2\pi k}{N}} = \cos\left(\frac{2\pi k}{N}\right) + i \sin\left(\frac{2\pi k}{N}\right), \tag{31}$$

for each $k \in \{0, 1, 2, \ldots, N{-}1\}$.

For a DFT of size N, the Fourier contour points are equal to the N complex roots of unity of order N. The ordering of the contour points matches the way in which the roots are enumerated. Also, the angle between two consecutive points is positive, which is in agreement with the right-hand rule.

The complex inner product between two complex vectors $\mathbf{a}$ and $\mathbf{b}$ of length N is defined as follows:

$$\langle \mathbf{a}, \mathbf{b} \rangle = \sum_{n=0}^{N-1} \mathrm{a}_n \cdot \overline{\mathrm{b}_n}\,. \tag{32}$$

Notice that the elements of the second vector are conjugated. Thus, changing the order of the two vectors in the inner product is equivalent to conjugating its value, i.e.,

$$\langle \mathbf{b}, \mathbf{a} \rangle = \overline{\langle \mathbf{a}, \mathbf{b} \rangle}. \tag{33}$$

This definition ensures that the complex inner product of a vector with itself is always nonnegative and is zero if and only if the vector is zero.

The DFT output vector $\mathbf{X}$ contains the values of N complex inner products between the DFT input vector $\mathbf{x}$ and each of the frequency component vectors $\mathbf{v}^{(0)}, \mathbf{v}^{(1)}, \mathbf{v}^{(2)}, \ldots, \mathbf{v}^{(N-1)}$. Each vector $\mathbf{v}^{(k)}$ is defined as follows:

$$\mathbf{v}^{(k)} = \left(\left(\mathrm{e}^{\frac{i2\pi k}{N}}\right)^0, \left(\mathrm{e}^{\frac{i2\pi k}{N}}\right)^1, \ldots, \left(\mathrm{e}^{\frac{i2\pi k}{N}}\right)^{N-1}\right). \tag{34}$$

Definition 4. ***Discrete Fourier Transform (DFT).***
Let $\mathbf{c} = (\mathrm{c}_0, \mathrm{c}_1, \mathrm{c}_2, \ldots, \mathrm{c}_{N-1})$ be the Fourier contour for a transform of size N. Let $\mathbf{x} = (\mathrm{x}_0, \mathrm{x}_1, \mathrm{x}_2, \ldots, \mathrm{x}_{N-1})$ be a complex input vector of length N. The DFT of the vector $\mathbf{x}$ is a complex vector $\mathbf{X}$, also of length N. For each $k \in \{0, 1, 2, \ldots, N{-}1\}$, the value of X_k is equal to the complex inner product between the vector $\mathbf{x}$ and the k-th frequency component vector $\mathbf{v}^{(k)}$. That is,

$$\mathrm{X}_k = \langle \mathbf{x}, \mathbf{v}^{(k)} \rangle = \sum_{n=0}^{N-1} \mathrm{x}_n \overline{\left(\mathrm{e}^{\frac{i2\pi k}{N}}\right)^n} = \sum_{n=0}^{N-1} \mathrm{x}_n\, \mathrm{e}^{-\frac{i2\pi kn}{N}}. \tag{35}$$

Figure S1 shows two different contours that can be derived from Eq. (35). Only the blue contour in Fig. S1a is consistent with Definition 3. The red contour in Fig. S1b is derived from the blue contour by conjugating all of its sampling points. This contour traverses the unit circle in the clockwise direction. The following color version of Eq. (35) visualizes the link between these two contours:

$$\mathrm{X}_k = \sum_{n=0}^{N-1} \mathrm{x}_n \overline{\left(\mathrm{e}^{\frac{i2\pi k}{N}}\right)^n} = \sum_{n=0}^{N-1} \mathrm{x}_n \left(\mathrm{e}^{-\frac{i2\pi k}{N}}\right)^n. \tag{36}$$

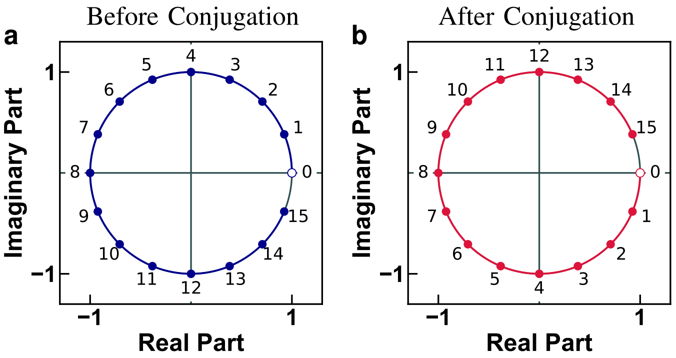

Fig. S1. Two 16-point contours based on two different interpretations of Eq. (35): (**a**) before the conjugation has been applied; and (**b**) after the conjugation has been applied. Only the contour in (**a**) is consistent with Definition 3.

Most references don't mention the conjugation and define the DFT using the rightmost sum in Eq. (35). Even though this is technically correct, it obscures the link to the Fourier contour and can lead to a lot of confusion. For example, without the conjugation it is more difficult to explain the essence of positive and negative frequencies and how the DFT/FFT orders them. Definition 4 makes things more clear by formulating the transform in terms of the complex inner product.

The IDFT inverts the DFT for the same contour. Definition 5 gives the formula for this inverse transform.

Definition 5. ***Inverse DFT (IDFT).***
The IDFT of a complex vector $\mathbf{X}$ of length N is a complex vector $\mathbf{x}$, also of length N. Its n-th element is given by:

$$\mathrm{x}_n = \frac{1}{N} \sum_{k=0}^{N-1} \mathrm{X}_k\, \mathrm{e}^{\frac{i2\pi kn}{N}}. \tag{37}$$

The following example illustrates the DFT of size 4. The elements of the output vector $\mathbf{X} = (\mathrm{X}_0, \mathrm{X}_1, \mathrm{X}_2, \mathrm{X}_3)$ are defined with the following four complex inner products:

$$\begin{aligned} \mathrm{X}_0 &= \langle \mathbf{x}, \mathbf{v}^{(0)} \rangle, \\ \mathrm{X}_1 &= \langle \mathbf{x}, \mathbf{v}^{(1)} \rangle, \\ \mathrm{X}_2 &= \langle \mathbf{x}, \mathbf{v}^{(2)} \rangle, \\ \mathrm{X}_3 &= \langle \mathbf{x}, \mathbf{v}^{(3)} \rangle. \end{aligned} \tag{38}$$

Using matrix notation, the output vector $\mathbf{X}$ can be expressed as the matrix–vector product between the DFT matrix $\mathbf{F}$ and the input vector $\mathbf{x}$, i.e., $\mathbf{X} = \mathbf{F}\,\mathbf{x}$. That is,

$$\underbrace{\begin{bmatrix} \mathrm{X}_0 \\ \mathrm{X}_1 \\ \mathrm{X}_2 \\ \mathrm{X}_3 \end{bmatrix}}_{\mathbf{X}} = \underbrace{\begin{bmatrix} 1 & 1 & 1 & 1 \\ 1 & \omega & \omega^2 & \omega^3 \\ 1 & \omega^2 & \omega^4 & \omega^6 \\ 1 & \omega^3 & \omega^6 & \omega^9 \end{bmatrix}}_{\mathbf{F}} \underbrace{\begin{bmatrix} \mathrm{x}_0 \\ \mathrm{x}_1 \\ \mathrm{x}_2 \\ \mathrm{x}_3 \end{bmatrix}}_{\mathbf{x}}, \tag{39}$$

where $\omega = \overline{\mathrm{e}^{\frac{i 2\pi}{N}}} = \mathrm{e}^{-\frac{i 2\pi}{N}}$.

Each row of the matrix $\mathbf{F}$ can be obtained by conjugating all elements of the corresponding frequency component vector defined in Eq. (34). That is,

$$\mathbf{F} = \begin{bmatrix} \overline{\mathrm{v}_0^{(0)}} & \overline{\mathrm{v}_1^{(0)}} & \overline{\mathrm{v}_2^{(0)}} & \overline{\mathrm{v}_3^{(0)}} \\ \overline{\mathrm{v}_0^{(1)}} & \overline{\mathrm{v}_1^{(1)}} & \overline{\mathrm{v}_2^{(1)}} & \overline{\mathrm{v}_3^{(1)}} \\ \overline{\mathrm{v}_0^{(2)}} & \overline{\mathrm{v}_1^{(2)}} & \overline{\mathrm{v}_2^{(2)}} & \overline{\mathrm{v}_3^{(2)}} \\ \overline{\mathrm{v}_0^{(3)}} & \overline{\mathrm{v}_1^{(3)}} & \overline{\mathrm{v}_2^{(3)}} & \overline{\mathrm{v}_3^{(3)}} \end{bmatrix} = \begin{bmatrix} 1 & 1 & 1 & 1 \\ 1 & -i & -1 & i \\ 1 & -1 & 1 & -1 \\ 1 & i & -1 & -i \end{bmatrix}. \tag{40}$$

The CZT also uses a contour, but its points are no longer restricted to lie on the unit circle. Its shape and winding direction are defined by the parameters A, W, and M.

Definition 6. ***Chirp contour.***
Let A and W be two complex numbers and let M be a positive integer. A chirp contour consists of M complex points $\mathrm{z}_0, \mathrm{z}_1, \mathrm{z}_2, \ldots, \mathrm{z}_{M-1}$ that lie on a logarithmic spiral. The starting point, z_0, is equal to A. The remaining points are derived by multiplying the parameter A with the negative integer powers of the parameter W. That is,

$$\mathrm{z}_k = A\,W^{-k}, \qquad \text{for each } k \in \{0, 1, 2, \ldots, M{-}1\}. \tag{41}$$

Each element X_k of the CZT output vector $\mathbf{X}$ is defined as the z-transform of the input vector $\mathbf{x}$, where the value of the z-transform is evaluated at the corresponding contour point z_k.

Definition 7. ***Chirp Z-Transform (CZT).***
Let $\mathbf{z} = (\mathrm{z}_0, \mathrm{z}_1, \mathrm{z}_2, \ldots, \mathrm{z}_{M-1})$ be the chirp contour. Let $\mathbf{x} = (\mathrm{x}_0, \mathrm{x}_1, \mathrm{x}_2, \ldots, \mathrm{x}_{N-1})$ be the complex input vector of length N. Each element X_k of the CZT complex output vector $\mathbf{X} = (\mathrm{X}_0, \mathrm{X}_1, \mathrm{X}_2, \ldots, \mathrm{X}_{M-1})$ is equal to the value of the z-transform of the input vector $\mathbf{x}$ at the corresponding contour point z_k. That is,

$$\mathrm{X}_k = \sum_{j=0}^{N-1} \mathrm{x}_j\, \mathrm{z}_k^{-j} = \sum_{j=0}^{N-1} \mathrm{x}_j (A\,W^{-k})^{-j} = \sum_{j=0}^{N-1} \mathrm{x}_j A^{-j}\, W^{jk}. \tag{42}$$

Similarly to the DFT, the CZT can also be defined using the complex inner product. In this case, the frequency component vectors are formed by conjugating the negative powers of the contour points. In other words, the CZT frequency components $\hat{\mathbf{v}}^{(0)}, \hat{\mathbf{v}}^{(1)}, \hat{\mathbf{v}}^{(2)}, \ldots, \hat{\mathbf{v}}^{(M-1)}$ can be expressed as follows:

$$\hat{\mathbf{v}}^{(k)} = \left(\overline{\left(A\,W^{-k}\right)^{-0}}, \overline{\left(A\,W^{-k}\right)^{-1}}, \ldots, \overline{\left(A\,W^{-k}\right)^{-(N-1)}} \right), \tag{43}$$

where $k \in \{0, 1, 2, \ldots, M{-}1\}$.

Each element X_k of the CZT output vector $\mathbf{X}$ can be expressed as the inner product between the CZT input vector $\mathbf{x}$ and the corresponding frequency component $\hat{\mathbf{v}}^{(k)}$. That is,

$$\begin{aligned} \mathrm{X}_k = \langle \mathbf{x}, \hat{\mathbf{v}}^{(k)} \rangle &= \sum_{j=0}^{N-1} \mathrm{x}_j\, \overline{\hat{\mathrm{v}}_j^{(k)}} = \sum_{j=0}^{N-1} \mathrm{x}_j\, \overline{\overline{\left(A\,W^{-k}\right)^{-j}}} \\ &= \sum_{j=0}^{N-1} \mathrm{x}_j \left(A\,W^{-k}\right)^{-j}. \end{aligned} \tag{44}$$

For the case when the CZT is equivalent to the DFT, i.e., $A = 1$, $W = \mathrm{e}^{-\frac{i 2\pi}{N}}$, and $M = N$, the frequency components of the CZT are equal to those of the DFT. That is,

$$\hat{\mathrm{v}}_j^{(k)} = \overline{\left(A\,W^{-k}\right)^{-j}} = \overline{\left(1 \cdot \mathrm{e}^{\frac{i 2\pi k}{N}}\right)^{-j}} = \mathrm{e}^{\frac{i 2\pi k j}{N}} = \mathrm{v}_j^{(k)}, \tag{45}$$

for each $k \in \{0, 1, \ldots, N{-}1\}$ and each $j \in \{0, 1, \ldots, N{-}1\}$. The chirp contour in this case matches the Fourier contour, e.g., for $M = 16$ it is equal to the blue contour in Fig. S1a.

Figure S2 gives an example with two chirp contours that lie on the unit circle. In both cases, the polar angle of A is positive and is equal to 30° and 45°, respectively. The contour shown in Fig. S2a rotates clockwise, because the polar angle of W is equal to 22.5°. The contour in Fig. S2b is drawn for the case when that angle is equal to −22.5°. This contour rotates counter-clockwise. These examples show that the right-hand rule is violated for the winding direction of chirp contours.

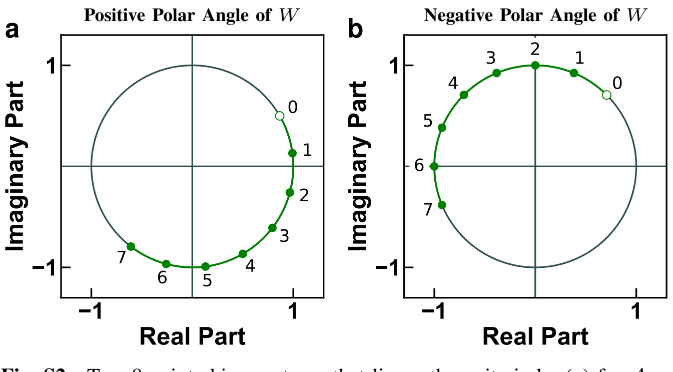

Fig. S2. Two 8-point chirp contours that lie on the unit circle: (**a**) for $A = \mathrm{e}^{\frac{i\pi}{6}}$ and $W = \mathrm{e}^{\frac{i\pi}{8}}$; and (**b**) for $A = \mathrm{e}^{\frac{i\pi}{4}}$ and $W = \mathrm{e}^{-\frac{i\pi}{8}}$. The contours are drawn according to Definition 6.

The chirp contour is shared by the CZT and the ICZT that have the same transform parameters. For the ICZT, however, there is no expression similar to Eq. (42) that uses the contour points to express the transform. Thus, for the ICZT it is more useful to view the contour as a visualization of the transform parameters A, W, and $M = N$.

S2. Mappings Between CZT, ICZT, FFT, and IFFT

This section shows how the CZT and ICZT algorithms can be used to compute the FFT and the IFFT for any N, i.e., not just a power of 2. Because orthogonal frequency components can be generated with either counter-clockwise or clockwise chirp contours, there are four possible algorithms. One of these mappings illustrates the ability to implement the IFFT using the CZT by reversing the contour direction and scaling the output vector. Some of the related work confused *reverse* with *inverse* and presented the resulting approach as the ICZT algorithm for all chirp contours that lie on the unit circle. The main paper shows why that approach is incorrect.

Algorithm S1 shows how to compute the FFT by calling the CZT algorithm with $M = N$, $W = \mathrm{e}^{-i2\pi/N}$, and $A = 1$. This works because these parameters define a Fourier contour with N points (see Section S1). Similarly, Algorithm S2 shows how to compute the IFFT by calling the ICZT. The parameter values in Algorithm S2 are the same as in Algorithm S1.

In contrast to the DFT, which uses a fixed contour that is traversed in only one direction, both the starting point and the contour direction can be varied with the CZT. This latter flexibility makes it possible to traverse the roots of unity in the clockwise direction and to compute the IFFT by calling the CZT. Algorithm S3 gives the pseudo-code for this approach. The CZT parameters in this case are $M = N$, $W = \mathrm{e}^{i2\pi/N}$, and $A = 1$. In addition, the algorithm scales all elements of the output vector by dividing them by N. This scaling is an integral part of the IFFT algorithm as well.

Similarly, the FFT can be implemented by calling the ICZT with $M = N$, $W = \mathrm{e}^{i2\pi/N}$, and $A = 1$. Again, the elements of the output vector need to be scaled. In this case, however, they are multiplied by N. Algorithm S4 gives the pseudo-code for this approach.

All four algorithms run in $O(n \log n)$, i.e., they have the same computational complexity as the standard FFT and IFFT algorithms. Their numerical accuracy, however, is somewhat lower than the numerical accuracy of the standard algorithms. The reason for this is that the CZT and the ICZT are more general algorithms that perform more operations, which increases the numerical error.

For the sake of completeness, Algorithm S5 gives the pseudo-code for the CZT algorithm. The pseudo-code for the ICZT is given in Algorithm 1. The dependencies for both algorithms are described in reference 10.

There is a peculiar extension of these mappings when the value of W is set to another *primitive* root of unity of order N instead of $\mathrm{e}^{-i2\pi/N}$ in Algorithms S1 and S2 or $\mathrm{e}^{i2\pi/N}$ in Algorithms S3 and S4. This leads to transforms that are still equivalent to the FFT or the IFFT after a cyclic permutation of the elements of the output vector.

The correspondence between these forward and inverse mappings breaks when the value of W is not a primitive root of unity of order N. For example, reversing the chirp contour computes the ICZT only if the frequency components are orthogonal, i.e., when the ICZT is equivalent to the IFFT after shuffling its output elements. This not so subtle issue was overlooked by some of the previous work. The main paper explains why this approach doesn't work.

Algorithm S1. FFT implemented using the CZT.

FFT-via-CZT($\mathbf{x}$)
$N \leftarrow$ Length($\mathbf{x}$);
$\mathbf{X} \leftarrow$ CZT($\mathbf{x}$, N, $\mathrm{e}^{-\frac{i2\pi}{N}}$, 1);
return $\mathbf{X}$;

Algorithm S2. IFFT implemented using the ICZT.

IFFT-via-ICZT($\mathbf{X}$)
$N \leftarrow$ Length($\mathbf{X}$);
$\mathbf{x} \leftarrow$ ICZT($\mathbf{X}$, N, $\mathrm{e}^{-\frac{i2\pi}{N}}$, 1);
return $\mathbf{x}$;

Algorithm S3. IFFT implemented using the CZT.

IFFT-via-CZT($\mathbf{X}$)
$N \leftarrow$ Length($\mathbf{X}$);
$\mathbf{x} \leftarrow$ CZT($\mathbf{X}$, N, $\mathrm{e}^{\frac{i2\pi}{N}}$, 1);
for $j \leftarrow 0$ **to** $N-1$ **do**
    $\mathrm{x}_j \leftarrow \frac{\mathrm{x}_j}{N}$;
end for
return $\mathbf{x}$;

Algorithm S4. FFT implemented using the ICZT.

FFT-via-ICZT($\mathbf{x}$)
$N \leftarrow$ Length($\mathbf{x}$);
$\mathbf{X} \leftarrow$ ICZT($\mathbf{x}$, N, $\mathrm{e}^{\frac{i2\pi}{N}}$, 1);
for $j \leftarrow 0$ **to** $N-1$ **do**
    $\mathrm{X}_j \leftarrow N \cdot \mathrm{X}_j$;
end for
return $\mathbf{X}$;

Algorithm S5. CZT Algorithm. Runs in $O(n \log n)$ time.

CZT($\mathbf{x}$, M, W, A)
$N \leftarrow$ Length($\mathbf{x}$);
$\mathbf{X} \leftarrow$ EmptyArray(N);
$\mathbf{r} \leftarrow$ EmptyArray(N);
$\mathbf{c} \leftarrow$ EmptyArray(M);
for $k \leftarrow 0$ **to** $N-1$ **do**
    $\mathrm{X}[k] \leftarrow W^{\frac{k^2}{2}} \cdot A^{-k} \cdot \mathrm{x}[k]$;
    $\mathrm{r}[k] \leftarrow W^{-\frac{k^2}{2}}$;
end for
for $k \leftarrow 0$ **to** $M-1$ **do**
    $\mathrm{c}[k] \leftarrow W^{-\frac{k^2}{2}}$;
end for
// After the next line, Length($\mathbf{X}$) $= M$.
$\mathbf{X} \leftarrow$ ToeplitzMultiplyE($\mathbf{r}$, $\mathbf{c}$, $\mathbf{X}$);
for $k \leftarrow 0$ **to** $M-1$ **do**
    $\mathrm{X}[k] \leftarrow W^{\frac{k^2}{2}} \cdot \mathrm{X}[k]$;
end for
return $\mathbf{X}$;

S3. Implementing ICTA and IFRFT using ICZT

The main paper mentioned two algorithms that generalize the *Fast Fourier Transform* (FFT) on the unit circle. They are the *Chirp Transform Algorithm* (CTA) and the *Fractional Fourier Transform* (FRFT) algorithm, which are described in references 15 and 16. The *Chirp Z-Transform* (CZT) is a generalization of the FFT off the unit circle. As described in this paper, however, the CZT can also be evaluated for circular chirp contours that lie on the unit circle. Thus, the CZT can also be viewed as a generalization of the FFT on the unit circle. Interestingly, both the CTA and the FRFT can be implemented with the CZT as shown below.

The corresponding inverse algorithms have not been discovered yet and have not been described in the literature until now. Both of these algorithms can be stated as special cases of the Inverse Chirp Z-Transform (ICZT) algorithm as described in this section. We took the liberty of naming these algorithms by adding an ‘I’ as the first letter in both acronyms.

Algorithm S6. Chirp Transform Algorithm (CTA).

1: $\mathrm{CTA}(\mathbf{x},\ M,\ \omega_0,\ \Delta\omega)$
2: $(W,\ A) \leftarrow (\mathrm{e}^{-i\Delta\omega},\ \mathrm{e}^{i\omega_0})$;
3: $\mathbf{X} \leftarrow \mathrm{CZT}(\mathbf{x},\ M,\ W,\ A)$;
4: **return** $\mathbf{X}$;

Algorithm S6 shows how to implement the CTA using the CZT. This is done by mapping the parameters of the CTA to the parameters of the CZT. The first two parameters are identical for both algorithms. They specify the input vector, $\mathbf{x}$, and the size of the output vector, M. The CTA defines the starting point of the contour using the starting angle ω_0. The angular distance between adjacent contour points is given by the parameter $\Delta\omega$. Line 2 of Algorithm S6 computes the CZT parameters W and A from the CTA parameters ω_0 and $\Delta\omega$. Line 3 calls the CZT algorithm to compute the output vector, which is returned on line 4. The pseudo-code for the CZT algorithm is given in Algorithm S5. Reference 10 gives the pseudo-code for all of its dependencies.

Historically, the chirp contour points were defined[7] using the z-transform instead of the power series (i.e., negative powers instead of positive powers, as described in Section S1). The CZT was also defined[7] using negative powers of the parameter A. More formally, the chirp contour points are set to the M complex numbers $\mathrm{z}_0, \mathrm{z}_1, \ldots, \mathrm{z}_{M-1}$ where $\mathrm{z}_k = A\,W^{-k}$. The value of the k-th element of the CZT output vector $\mathbf{X}$ is equal to the value of the z-transform at z_k of the CZT input vector $\mathbf{x}$, i.e.,

$$\mathrm{X}_k = \sum_{j=0}^{N-1} \mathrm{x}_j\, \mathrm{z}_k^{-j}, \quad \text{where } k \in \{0, 1, \ldots, M-1\}. \tag{46}$$

Following these traditions requires setting W to $\mathrm{e}^{-i\Delta\omega}$ and A to $\mathrm{e}^{i\omega_0}$ when mapping the CTA to the CZT. In other words, a positive value of ω_0 corresponds to a counter-clockwise offset for the starting point of the chirp contour relative to the point $(1, 0)$ on the unit circle. Similarly, a positive value of $\Delta\omega$ corresponds to a counter-clockwise winding direction of the chirp contour[15].

Algorithm S7. Fractional Fourier Transform (FRFT).

$\mathrm{FRFT}(\mathbf{x},\ m,\ \alpha)$
$(M,\ W,\ A) \leftarrow (m,\ \mathrm{e}^{-i2\pi\alpha},\ 1)$;
$\mathbf{G} \leftarrow \mathrm{CZT}(\mathbf{x},\ M,\ W,\ A)$;
return $\mathbf{G}$;

Algorithm S7 gives the pseudo-code for implementing the FRFT using the CZT. Once again, this is done by mapping the parameters of the FRFT to the parameters of the CZT. In this case, $\mathbf{x}$ is the input vector, which is the same for both algorithms. The second parameter is m, which is equivalent to M in the CZT formulation. The last parameter, α, specifies the angular distance between two adjacent contour points. It maps to the parameter W through the formula $W = \mathrm{e}^{-i2\pi\alpha}$. By definition, all FRFT contours start at 1. Thus, the CZT parameter A, which specifies the starting point of the contour, is always set to 1. The CTA is more flexible in this respect, because it allows the starting point of the contour to be any point on the unit circle, i.e., by varying the parameter ω_0.

Both the CTA and the FRFT can be used with contours that perform more than one revolution on the unit circle. Both algorithms run in $O(n \log n)$ time.

Algorithm S8. Inverse Chirp Transform Algorithm (ICTA).

$\mathrm{ICTA}(\mathbf{X},\ N,\ \omega_0,\ \Delta\omega)$
$(W,\ A) \leftarrow (\mathrm{e}^{-i\Delta\omega},\ \mathrm{e}^{i\omega_0})$;
$\mathbf{x} \leftarrow \mathrm{ICZT}(\mathbf{X},\ N,\ W,\ A)$;
return $\mathbf{x}$;

Algorithm S9. Inverse Fractional Fourier Transform (IFRFT).

$\mathrm{IFRFT}(\mathbf{G},\ m,\ \alpha)$
$(\mathbf{X},\ N,\ W,\ A) \leftarrow (\mathbf{G},\ m,\ \mathrm{e}^{-i2\pi\alpha},\ 1)$;
$\mathbf{x} \leftarrow \mathrm{ICZT}(\mathbf{X},\ N,\ W,\ A)$;
return $\mathbf{x}$;

Algorithms S8 and S9 show how to implement the ICTA and the IFRFT by mapping their parameters to the ICZT parameters and then calling Algorithm 1. Algorithm S8 computes the values of W and A for the ICZT from the parameters ω_0 and $\Delta\omega$ and then calls Algorithm 1 to compute the transform. Similarly, Algorithm S9 computes the ICZT parameters from $\mathbf{G}$, m, and α and then also calls Algorithm 1. Both algorithms run in $O(n \log n)$ time and use $O(n)$ memory.

By definition, the ICTA and the IFRFT algorithms can work only with contours that lie on the unit circle. The ICZT algorithm, however, can also work with logarithmic spiral contours that lie off the unit circle[10]. Thus, the ICZT is more general than both the ICTA and the IFRFT.

S4. The Rank of the Matrix $\boldsymbol{W}$ Depends on the Polar Angle of the Transform Parameter W

This section provides additional proofs that complement the results from Theorem 2. In this case, the analysis is performed using the rank of the matrix $\boldsymbol{W}$ in Eq. (4) instead of the singularities of Eq. (11) that specifies the generating vector $\mathbf{u}$.

Theorem 3. *Let p/q be a rational number represented as an irreducible fraction, i.e., p is an integer and q is a positive integer such that $\gcd(p,q) = 1$. Furthermore, let n be a positive integer. Then, the number of elements in the set $S = \left\{ e^{i2\pi \frac{p\cdot 0}{q}}, e^{i2\pi \frac{p\cdot 1}{q}}, \dots, e^{i2\pi \frac{p\cdot (n-1)}{q}} \right\}$ is equal to the smaller of q and n. More formally,*

$$\left| \left\{ e^{i2\pi \frac{pk}{q}},\ k \in \{0,1,\dots,n-1\} \right\} \right| = \min(q,n). \tag{47}$$

Proof. Let k_1 and k_2 be two different integers that lie between 0 and $\min(q,n)-1$. That is,

$$k_1, k_2 \in \{0,1,2,\dots,\min(q,n)-1\}, \quad k_1 \neq k_2. \tag{48}$$

Then, the difference between their corresponding elements in the set S can be expressed as follows:

$$e^{i2\pi \frac{p}{q} k_1} - e^{i2\pi \frac{p}{q} k_2} = e^{i2\pi \frac{p}{q} k_1}\left(1 - e^{i2\pi \frac{p}{q}(k_2-k_1)}\right). \tag{49}$$

Without loss of generality, suppose that $k_1 < k_2$. Then, the following inequality holds:

$$1 \leq k_2 - k_1 \leq \min(q,n) - 1 < q. \tag{50}$$

Because $\gcd(p,q) = 1$, it follows that p and q share no prime factors. Therefore,

$$\gcd(p(k_2-k_1), q) = \gcd(k_2-k_1, q) \leq k_2 - k_1 < q. \tag{51}$$

That is, each prime factor shared by the product $p(k_2-k_1)$ and q must be shared by $k_2 - k_1$ and q. Moreover, the value of $\gcd(k_2-k_1,q)$ can't exceed $k_2 - k_1$, which is strictly less than q. This implies that the fraction $\frac{p(k_2-k_1)}{q}$ is not reducible to an integer. Hence,

$$e^{i2\pi \frac{p}{q}(k_2-k_1)} \neq 1, \tag{52}$$

which implies that:

$$e^{i2\pi \frac{p}{q} k_1} \neq e^{i2\pi \frac{p}{q} k_2}. \tag{53}$$

Therefore, the mapping from $k \in \{0,1,2,\dots,\min(q,n)-1\}$ to $e^{i2\pi \frac{p}{q} k}$ is one-to-one. Thus, there are at least $\min(q,n)$ distinct elements in the set S, which proves that $|S| \geq \min(q,n)$.

To prove the equality in Eq. (47), it only remains to show that $|S| \leq \min(q,n)$. There are two possible cases: $n \leq q$ and $n > q$. If $n \leq q$, then $|S| \leq n = \min(q,n)$ because, by definition, the number of elements in S cannot exceed n.

If $n > q$, then for each $k \geq q$ the value of $e^{i2\pi \frac{p}{q} k}$ is equal to $e^{i2\pi \frac{p}{q}(k-q)}$. In other words, the elements begin to repeat starting with $k = q$. More formally,

$$e^{i2\pi \frac{p(k-q)}{q}} = e^{i2\pi \frac{pk}{q}} \underbrace{e^{-i2\pi p}}_{1} = e^{i2\pi \frac{pk}{q}}, \tag{54}$$

for each $k \geq q$. Thus, $|S| \leq q = \min(q,n)$. □

Theorem 4. *Let p/q be a rational number that is represented using an irreducible fraction, i.e., p is an integer and q is a positive integer such that $\gcd(p,q) = 1$. Let M and N be two positive integers. Also, let $\boldsymbol{W}$ be the M-by-N Vandermonde matrix used by the CZT where $W = e^{i2\pi p/q}$. That is,*

$$\boldsymbol{W} = \begin{bmatrix} W^{0\cdot 0} & W^{1\cdot 0} & \dots & W^{(N-1)\cdot 0} \\ W^{0\cdot 1} & W^{1\cdot 1} & \dots & W^{(N-1)\cdot 1} \\ \vdots & \vdots & \ddots & \vdots \\ W^{0\cdot (M-1)} & W^{1\cdot (M-1)} & \dots & W^{(N-1)\cdot (M-1)} \end{bmatrix}. \tag{55}$$

Then,

$$\operatorname{rank}(\mathbf{W}) = \min(q,n), \quad \textit{where } n = \min(M,N). \tag{56}$$

Proof. Without loss of generality, suppose that $M \leq N$, which implies that $n = M$ (otherwise, if $M > N$, then the matrix $\boldsymbol{W}$ can be replaced with its transpose, which doesn't affect the rank). The matrix $\boldsymbol{W}$ is a Vandermonde matrix that is generated by the vector $\mathbf{s} = (W^0, W^1, W^2, \dots, W^{n-1})$. The number of distinct elements in this vector is equal to $\min(q,n)$, which follows from Theorem 3. Thus, the number of distinct rows in the matrix $\boldsymbol{W}$ is also equal to $\min(q,n)$. This implies that $\operatorname{rank}(\boldsymbol{W}) \leq \min(q,n)$.

To prove the equality in Eq. (56) we will show that the first r rows of $\boldsymbol{W}$ are linearly independent, where $r = \min(q,n)$. Let $\boldsymbol{V}$ be a square sub-matrix of $\boldsymbol{W}$ that lies in the intersection of its first r rows and r columns. The matrix $\boldsymbol{V}$ is also a Vandermonde matrix that is generated by the vector $(W^0, W^1, W^2, \dots, W^{r-1})$. Theorem 3 proved that the number of distinct elements in this vector is equal to r.

The determinant of $\boldsymbol{V}$ is given by the following formula (see reference 39, p. 191 or reference 43, p. 78):

$$\det(\boldsymbol{V}) = \prod_{0 \leq i < j \leq n-1} (W^j - W^i) \neq 0. \tag{57}$$

This implies that the matrix $\boldsymbol{V}$ is non-singular, i.e, that it has full rank. More formally, $\operatorname{rank}(\boldsymbol{V}) = r$. Because $\boldsymbol{V}$ is a sub-matrix of $\boldsymbol{W}$, it follows that

$$r = \operatorname{rank}(\boldsymbol{V}) \leq \operatorname{rank}(\boldsymbol{W}). \tag{58}$$

Thus, Eq. (56) holds because $r \leq \operatorname{rank}(\boldsymbol{W}) \leq r$. □

The following theorem reinterprets Theorem 4 in terms of Farey sequences. It shows that the matrix $\boldsymbol{W}$ is singular when the polar angle of the transform parameter W is a Farey angle of order $n-1$, where $n = M = N$ is the size of the transform.

Theorem 5. *The matrix $\mathbf{W}$ is singular when $p/q \in F_{n-1}$, i.e., p/q is a member of the Farey sequence of order $n-1$. Conversely, if $p/q \notin F_{n-1}$, then $\mathbf{W}$ is non-singular. Another way to state these conditions is: $\mathbf{W}$ is singular when $q < n$ and nonsingular when $q \geq n$, where q is a positive integer, $p \in \{0,1,\dots,q\}$, $p/q \in [0,1]$, $\gcd(p,q) = 1$, and $W = e^{i2\pi p/q}$.*

Proof. The definition of a Farey sequence implies that all rational numbers p/q between 0 and 1 for which $q < n$ are elements of F_{n-1}. Thus, $\mathbf{W}$ is singular whenever $p/q \in F_{n-1}$. Conversely, if $p/q \notin F_{n-1}$, then $q \geq n$ and $\min(q,n) = n$, which implies that the matrix $\boldsymbol{W}$ is non-singular. □

Theorem 6. *Let F_n be the Farey sequence of order n. Then, the sequence $S = \left\{ e^{i2\pi p/q} : p/q \in F_n \right\}$ lists each complex root of unity of order $1, 2, \ldots, n$ exactly once. Let Ω be the set of these roots of unity. More formally,*

$$\Omega = \{\omega_b^a \ : \ b \in \{1, 2, \ldots, n\}, \ a \in \{0, 1, 2, \ldots, b-1\}\}, \tag{59}$$

where $\omega_b^a = e^{i2\pi a/b}$. Then, $\Omega = S$.

Proof. Let p/q be an element of F_n. Then, $q \in \{1, 2, \ldots, n\}$, and $p \in \{0, 1, \ldots, q\}$, which implies $(e^{i2\pi p/q})^q = e^{i2\pi p} = 1$. Thus, $e^{i2\pi p/q}$ is a q-th root of unity. Therefore, $e^{i2\pi p/q} \in \Omega$. From this it follows that $S \subseteq \Omega$.

Conversely, let $\omega_b^a \in \Omega$. Then, $\omega_b^a = e^{i2\pi a/b}$, where $a \in \{0, 1, 2, \ldots, b-1\}$ and $b \in \{1, 2, \ldots, n\}$. Let p and q be two integers defined as: $p = a/\gcd(a, b)$ and $q = b/\gcd(a, b)$. Because $\gcd(a, b) \geq 1$, it follows that $1 \leq q \leq b \leq n$. Because $a < b$ and p and q are obtained by dividing a and b with the same positive number, it follows that $0 < p < q \leq n$. Thus, $p/q \in F_n$. Therefore, $e^{i2\pi a/b} = \omega_b^a = e^{i2\pi p/q} \in S$, from which it follows that $\Omega \subseteq S$.

Combining the two results leads to $\Omega = S$. □

The next theorem proves that using a chirp contour with sampling points that coincide with the sampling points used by the FFT, i.e., a chirp contour with $W = e^{i2\pi k/n}$ where k is coprime with n, never leads to singularities in the CZT matrix. This is done by showing that, in this special case, the fraction k/n appears for the first time in the Farey sequence F_n. The singularities of the CZT matrix, however, are determined by the elements of the preceding Farey sequence F_{n-1}. Thus, the singularities are avoided when the CZT reduces to the FFT. The same argument applies to the ICZT and the IFFT.

Theorem 7. *Let n be a positive integer and let $\omega_n^k = e^{i2\pi \frac{k}{n}}$ be a* <u>*primitive*</u> *root of unity of order n, i.e., ω_n^k is not a root of unity of any order smaller than n. Then, the following two conditions hold:*

1) *the fraction k/n is an element of the Farey sequence of order n, i.e,. $k/n \in F_n$;*
2) *the fraction k/n is not an element of the Farey sequence of order $n-1$, i.e., $k/n \notin F_{n-1}$.*

Proof. Without loss of generality, we can assume that k is an integer between 0 and $n-1$ (otherwise, if $k \geq n$ or $k < 0$, then we can set k to $k - \left\lfloor \frac{k}{n} \right\rfloor n$, which is between 0 and $n-1$, without changing the value of $e^{i2\pi k/n}$). Therefore, by definition, the fraction k/n is an element of the Farey sequence of order n, i.e., $k/n \in F_n$.

The second condition is proven by contradiction. Suppose that $k/n \in F_{n-1}$. This implies that the fraction k/n is reducible, i.e., $k/n = p/q$, where q is a positive integer smaller than n and p is an integer between 0 and $q-1$. In turn, this reducibility implies that ω_n^k is not a *primitive* root of unity of order n because it is a root of unity of order q where $q < n$. That is,

$$(\omega_n^k)^q = e^{i2\pi \frac{k}{n} q} = e^{i2\pi \frac{p}{q} q} = e^{i2\pi p} = 1. \tag{60}$$

This contradiction proves that $k/n \notin F_{n-1}$, as required. □

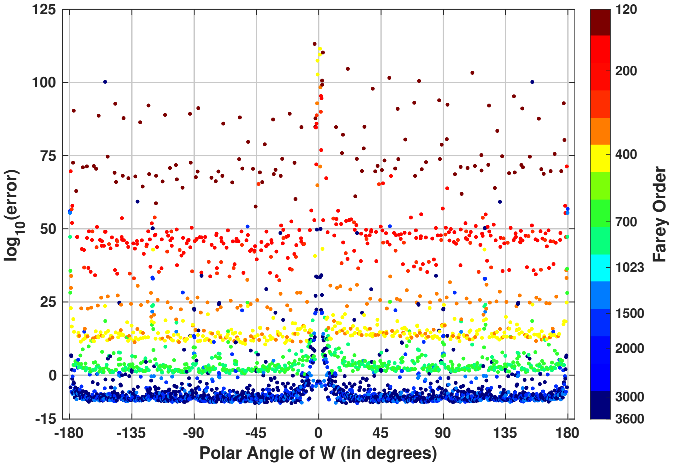

Fig. S3. Explanation of the layering in Fig. 6a in terms of the Farey order of each angle used in this discretization.

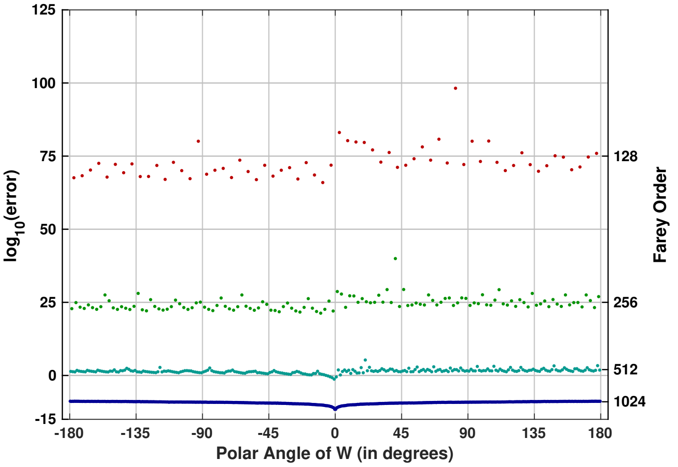

Fig. S4. Another example with color-coded layers, this time from Fig. 7a, where both the transform size and the number of regularly-discretized polar angles were set to 1024.

Figure S3 illustrates Theorem 4 and Theorem 5. It shows the same plot as in Fig. 6a but also colors each point based on the Farey order of its corresponding polar angle of W. The colors nicely explain the stratification of the error function for this discretization, which uses a step of $0.1°$. The upper layers, which have very large errors, correspond to lower Farey orders. The lower layers correspond to larger Farey orders. In general, the transform is numerically accurate for Farey orders that exceed or are equal to the transform size. In this plot they are drawn with blue colors.

Figure S4 gives another illustration of these effects using a color version of the plot shown in Fig. 7a. In this case the Farey orders can only be integer powers of two, because both the number of regularly-discretized angles and the transform size are equal to 1024. The four layers in this plot correspond to Farey orders 128, 256, 512, and 1024. The numerical error for Farey orders less than 128 is too large for computations with double precision, leading to either `NaN`[25] or infinite numerical error values for those polar angles of W.

S5. Additional Results for 32-Point Chirp Contours

This section studies the behavior of the numerical error for 32-point chirp contours on the unit circle. The goal is to determine if the harmonically spaced spiking pattern shown in Fig. 5a (i.e., the "harmonic hedgehog") can be observed for these contours as well.

Figure S5 plots the absolute numerical error for 32-point contours as a function of the polar angle of the transform parameter W. The angles were discretized using a 0.1° step. The overall shape of the numerical error function is similar to the error shown in Fig. 4, but the pattern becomes more complicated. For example, the features at the bottom of the figure are less distinct at this resolution and appear to be compressed by a factor of 2.

Figure S6 shows a close-up view of the numerical error for angles between 10° and 23°. The discretization interval in this case is 0.005°. The figure reveals another spiking pattern. In this case, however, there are 16 spikes instead of 8. Also, the Farey angles in this case are related to F_{31} instead of F_{15}. Furthermore, the harmonic hedgehog is now squeezed in the interval $[11°, 23°]$ instead of the interval $[22°, 46°]$ as in Fig. 5a. In addition to the discretized angles, the plot also includes points that correspond to Farey angles in the set $\{\frac{360°}{31}, \frac{360°}{30}, \frac{360°}{29}, \ldots, \frac{360°}{16}\}$. The vertical coordinate exceeds zero only for these sixteen Farey angles.

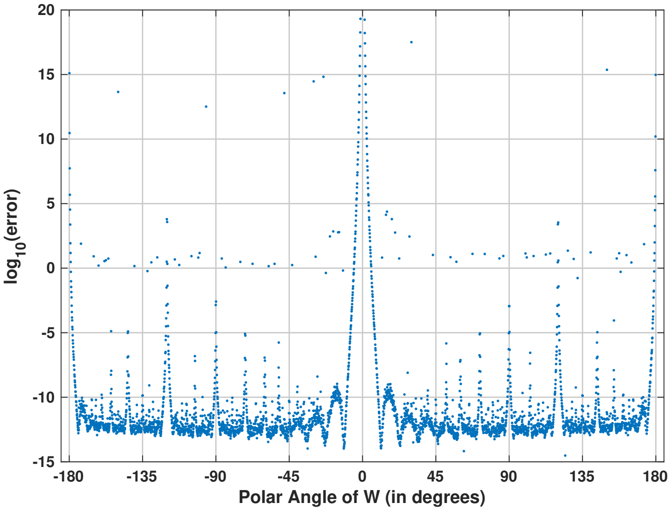

Fig. S5. Absolute numerical error of the CZT–ICZT procedure for chirp contours with 32 points on the unit circle. The discretization step for the polar angles of W was set to 0.1°, which resulted in 3600 angles.

The red points in Fig. S6 correspond to chirp contours with polar angles equal to 22.2°, 22.5°, and 22.8° (see Fig. S7). The middle red point coincides with a spike in the numerical error because its corresponding contour has only 16 distinct sampling points. That is, the contour makes exactly 2 revolutions and the first 16 points coincide with the second 16 points. The CZT transformation matrix for this contour is singular, i.e., it is not invertible. Section S4 proves that the matrix $\boldsymbol{W}$ (see Eq. (4)) is not full rank when the polar angle of W is a Farey angle of order q and q is strictly less than the transform size n. In this case, $q = 16$ and $n = 32$, which explains why the error spikes near this angle in Fig. S6.

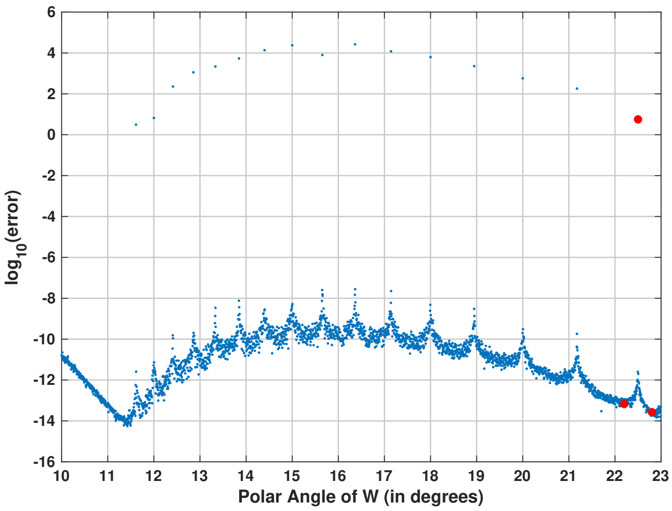

Fig. S6. The harmonic hedgehog for chirp contours with 32 points has 16 spikes. The absolute numerical error spikes when the polar angle of W is close to an element of the set $\{\frac{360°}{31}, \frac{360°}{30}, \frac{360°}{29}, \ldots, \frac{360°}{16}\}$. The three red points indicate the error for each of the three contours shown in Fig. S7.

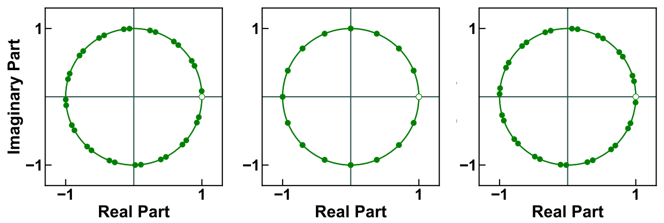

Fig. S7. Three chirp contours, each with 32 points, that wrap twice around the unit circle. From left to right, the polar angle of W is: 22.2°, 22.5°, and 22.8°. The middle contour has only 16 distinct points because 22.5° is equal to $360°/16$. In other words, the 16 points from the second revolution coincide with the 16 points from the first revolution.

The error for the other two contours from Fig. S7 is relatively small. Each of them has 16 pairs of points that are very close to each other but do not coincide. The left contour corresponds to a Farey angle of order 600. The right contour corresponds to a Farey angle of order 300. In contrast, the middle contour corresponds to a Farey angle of order 16, which is below the transform size that is equal to 32.

Once again, the results indicate that the numerical error is very small for most contours that were tried. The only exceptions are contours for which the polar angle of W is close to a Farey angle or 0°, which is also a Farey angle. In theory, Theorem 2 from the main paper suggests that the error should be infinite when the polar angle of W is a Farey angle of order less than n. In practice, however, the error is often large but finite for these angles. For example, even though sixteen Farey angles were explicitly added in Fig. S6, the absolute numerical error for these points is still between 10^0 and 10^5. This is due to the inability of the IEEE-754 floating-point representation[25], which is used by modern computers, to represent these Farey angles and their complex exponentials exactly.

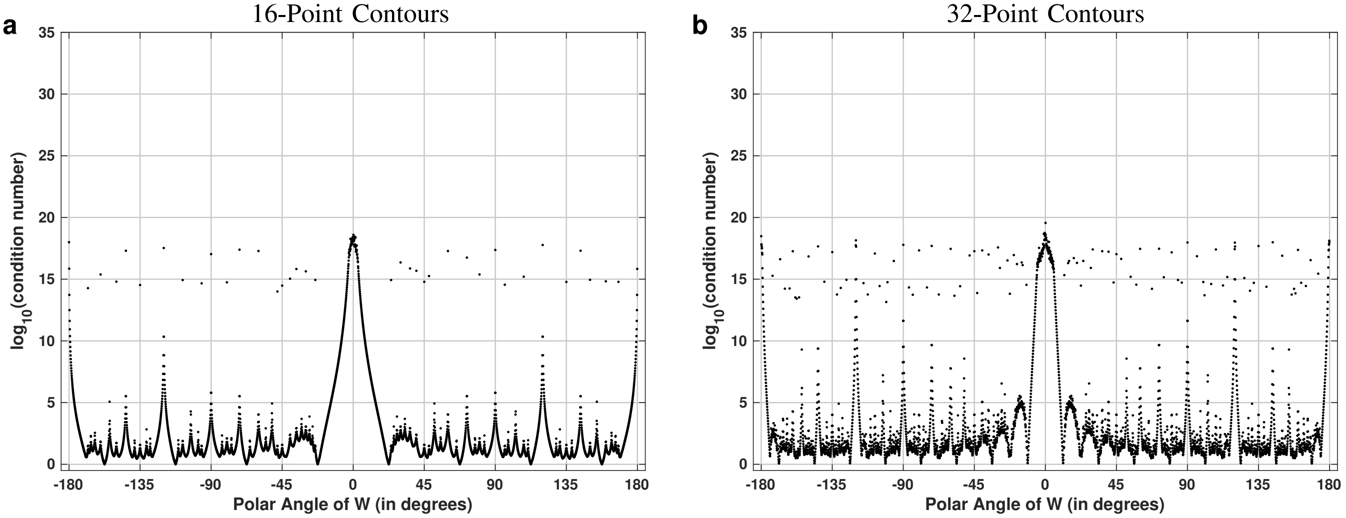

Fig. S8. Condition numbers for the transform matrix $\boldsymbol{W}$, shown as a function of the polar angle of the transform parameter W for chirp contours on the unit circle with 16 points in (**a**) and 32 points in (**b**). For both plots, the polar angles were discretized at 0.1° intervals, for a total of 3600 regularly-spaced angles.

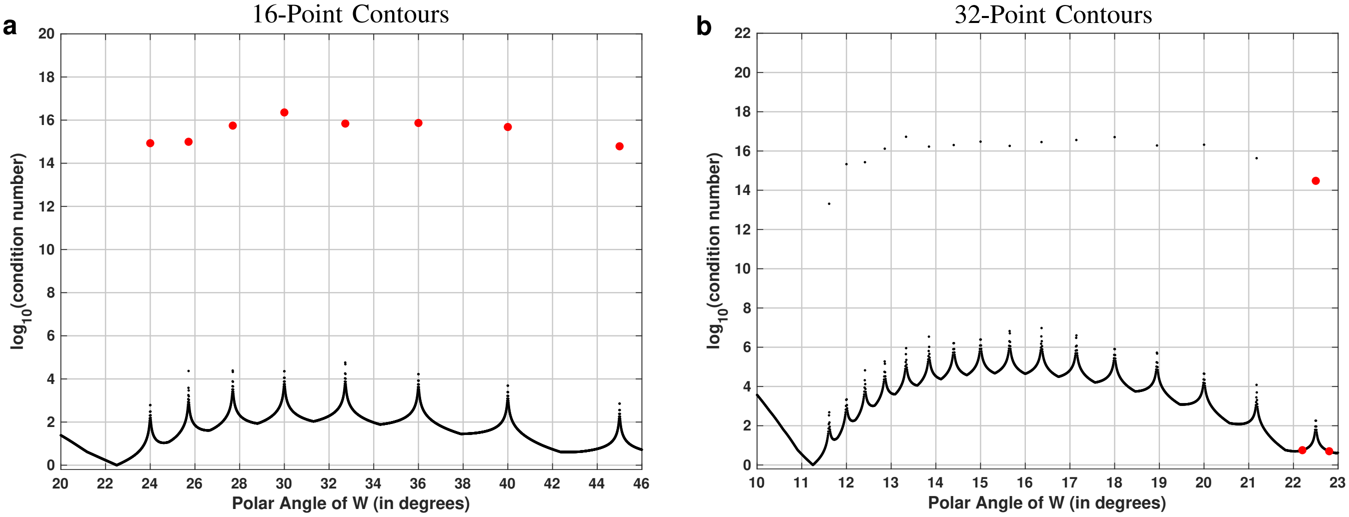

Fig. S9. (**a**) Close-up of the harmonic hedgehog between 20° and 46° in Fig. S8a. The eight red points indicate the condition numbers at the Farey angles in the set $\{\frac{360^\circ}{15}, \frac{360^\circ}{14}, \frac{360^\circ}{13}, \ldots, \frac{360^\circ}{8}\}$. (**b**) Close-up of the harmonic hedgehog between 10° and 23° in Fig. S8b. The three red points indicate the condition numbers for each of the three contours from Fig. S7. The condition number spikes near Farey angles in the set $\{\frac{360^\circ}{31}, \frac{360^\circ}{30}, \frac{360^\circ}{29}, \ldots, \frac{360^\circ}{16}\}$. The shapes of these plots are similar to the shapes of the numerical error plots in Fig. 5a and Fig. S6, respectively.

S6. Condition Numbers for 16-Point and 32-Point Chirp Contours

Figure S8 plots the condition number of the matrix $\boldsymbol{W}$ from Eq. (4) as a function of the polar angle of the parameter W for 16-point and 32-point contours. For both plots, the discretization used 3600 regularly-spaced polar angles between 0° and 360°, i.e., in increments of 0.1°. The condition numbers were computed with 64-bit floating-point numbers as follows: $\text{cond}(\boldsymbol{W}) = \sigma_{\max}/\sigma_{\min}$, where $\sigma_{\max}$ is the maximum and $\sigma_{\min}$ is the minimum singular value of the matrix $\boldsymbol{W}$.

For 16-point contours, the shape of the condition number plot in Fig. S8a is similar to the shapes of the predicted and empirical numerical error plots shown in Fig. 4 in the main paper. For 32-point contours, the shape of the plot in Fig. S8b is similar to the shape of the numerical error plot in Fig. S5. The locations of the peaks in Figure S8 are in agreement with the theoretical results from Section S4.

Figure S9 shows close-ups of two harmonic hedgehogs. Fig. S9a zooms in on the interval $[20^\circ, 46^\circ]$ from Fig. S8a. Fig. S9b focuses on the interval $[10^\circ, 23^\circ]$ from Fig. S8b. In both plots, the discretization interval is 0.005°.

The red points in Fig. S9a indicate the condition numbers for the Farey angles that correspond to the elements of the Farey sequence F_{15} between $1/15$ and $1/8$. These angles were explicitly added because they were missed by this discretization. Similarly, the points that correspond to the elements of F_{31} between $1/31$ and $1/16$ were included in Fig. S9b. The three red points in that figure indicate the condition numbers for the three 32-point contours from Fig. S7.

The shapes of the two harmonic hedgehogs in Fig. S9 are similar to the numerical error plots in Fig. 5a and Fig. S6. This suggests that the pattern of harmonically-spaced spikes can be observed not only with the numerical error but also with the condition number for this problem.

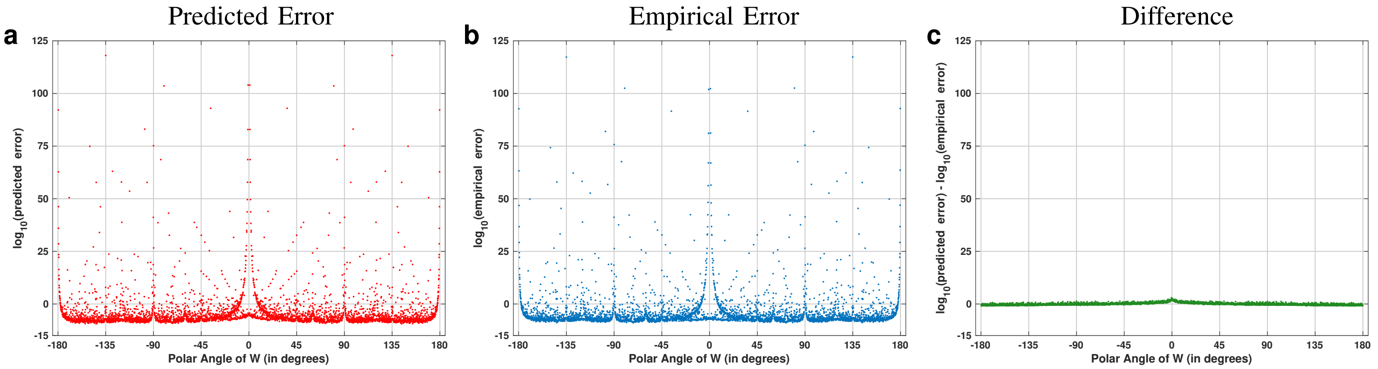

Fig. S10. Visualization of the predictive accuracy of Eq. (21) for 2048-point chirp contours. The red points in (**a**) show the predicted numerical error for the CZT followed by the ICZT, computed for 4099 regularly-discretized polar angles of W. The blue points in (**b**) show the empirically-observed numerical error, which is averaged over 10 random input vectors. The green points in (**c**) show the difference between the predicted error and the empirically observed error, i.e., (**a**) $-$ (**b**).

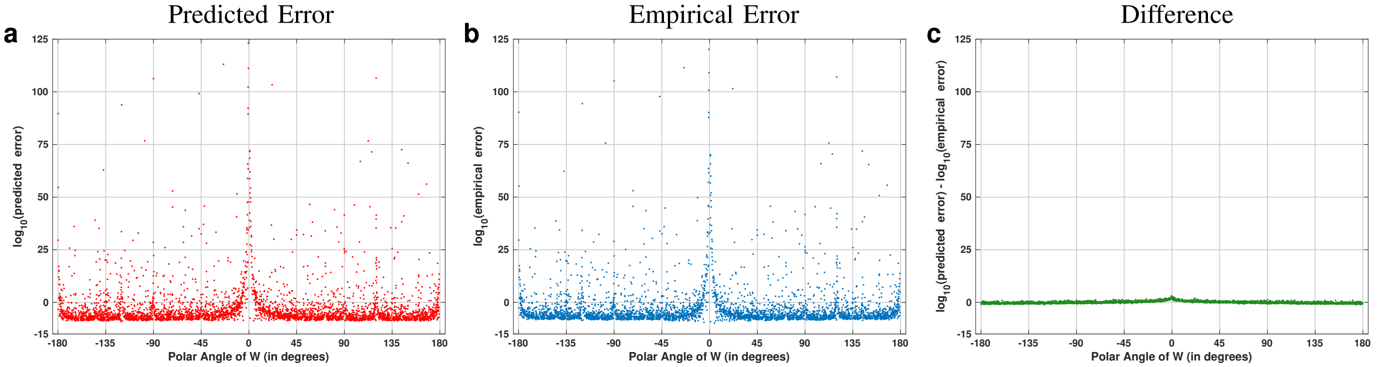

Fig. S11. Visualization of the accuracy of the error prediction formula for the CZT followed by the ICZT. These are similar to the plots in Fig. S10, but in this case the results are shown for 4099 randomly-sampled polar angles of W.

S7. Additional Error Analysis Results

Figure S10 visualizes the accuracy of the numerical error predictions obtained with Eq. (21). The results are plotted for 2048-point chirp contours and for 4099 regularly-discretized polar angles of W. The red points in Fig. S10a show the predicted numerical error. The blue points in Fig. S10b show the average empirically-observed error. The green points in Fig. S10c show the difference between the predicted error and the observed error, i.e, red minus blue. Figure S11 shows a similar analysis for randomly-sampled polar angles.

Figures S10c and S11c plot the difference between the logarithms of the predicted numerical error and the empirical numerical error. In both cases, the predicted error is close to the empirical error, which is reflected in the green horizontal line at zero. The slight bump around $0°$ in both figures suggests that the error prediction formula slightly overestimates the numerical error for polar angles of W that are close to $0°$. Because the R^2 coefficient tends to 1 as additional points are added to the chirp contour, the impact of this overestimation effect relative to the total variance of the numerical error diminishes as N increases.

For discretizations that hit many Farey angles, both the predicted and the observed errors could be very large (e.g., see Fig. 6a). For some of these singularities, the formulas described in the main paper underestimate the error. Section S8 analyzes these special cases in more detail and shows how to patch the numerical error prediction formulas by modifying the offset term B when the value of W is close to an ICZT singularity. This change makes the predictions more accurate for these extreme cases, but those values of W should not be used in practice because even the original formulas predict a very large error. This patch was not used for Figs. S10 and S11.

All algorithms described in this paper were implemented in C++. For this implementation, the constants C_1 and C_2 in Eq. (19) have the following values: $C_1 = 1$ and $C_2 = -1$. In our previous paper[10], the algorithms were implemented in Python using the *mpmath* library (see http://mpmath.org/), which slightly boosts the precision of complex exponentiation. There the precision was further boosted when computing all generating vectors for the matrices used by the CZT and the ICZT. This further decreased the numerical error so that the value of C_1 for that implementation reduced to -1 and the value of C_2 became 0. These implementation-specific details affect only the offset term B, i.e., they only shift the error function up or down by a fixed amount without affecting its overall shape.

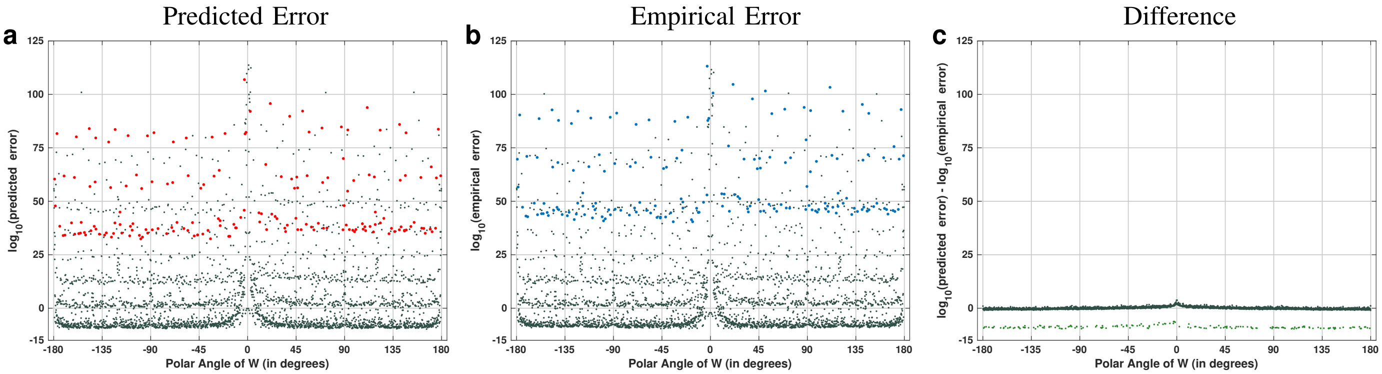

Fig. S12. Example in which the numerical error is sometimes underestimated. The predicted numerical error is shown in (**a**). The empirically-observed error is shown in (**b**) – this is the same plot as in Fig. 6a. The difference between (**a**) and (**b**) is shown in (**c**). The green points in (**c**) indicate all cases for which Eq. (19) and Eq. (21) underestimate the numerical error. The red points in (**a**) and the blue points in (**b**) show the predicted and the empirically-observed errors for these green points.

S8. Error Analysis Near ICZT Singularities

This section describes a slight modification for the error prediction formulas that makes them more accurate near ICZT singularities. The formulas described in the main paper can underestimate the predicted error for some of these cases. More specifically, the offset term B given by Eq. (19), which is used in Eq. (21) and Eq. (23), is modified here to be more accurate near ICZT singularities. This modification is not necessary in practice because even the underestimated error value is already very high.

Figure S12 shows an example for a regular discretization with a step of 0.1° that hits many singularities. For some of them the predicted numerical error is underestimated, as indicated by the green points below zero in Fig. S12c. The empirically-observed errors for these green points are highlighted in blue in Fig. S12b. The corresponding predicted errors are highlighted in red in Fig. S12a. The error is underestimated because the red points appear lower than the blue points.

The modified offset term B is computed using three helper terms: S_1, S_2, and S_3. The terms S_1 and S_2 are equal to the logarithms of the norms of the vectors $\mathbf{x}'$ and $\mathbf{x}''$ that are computed by Algorithm 1 on lines 25–28, i.e.,

$$S_1 = \log \|\mathbf{x}'\| = \log \sqrt{\sum_{k=0}^{N-1} |\mathrm{x}'_k|^2} = \frac{1}{2} \log \sum_{k=0}^{N-1} |\mathrm{x}'_k|^2, \quad (61)$$

$$S_2 = \log \|\mathbf{x}''\| = \log \sqrt{\sum_{k=0}^{N-1} |\mathrm{x}''_k|^2} = \frac{1}{2} \log \sum_{k=0}^{N-1} |\mathrm{x}''_k|^2. \quad (62)$$

The term S_3 is equal to the logarithm of the Euclidean distance between $\mathbf{x}'$ and $\mathbf{x}''$. That is,

$$S_3 = \log \|\mathbf{x}' - \mathbf{x}''\| = \frac{1}{2} \log \sum_{k=0}^{N-1} |\mathrm{x}'_k - \mathrm{x}''_k|^2. \quad (63)$$

Near ICZT singularities the difference between S_3 and the larger value between S_1 and S_2 captures the numerical error offset better than the constant term $-p \log 2$ used in Eq. (19).

Let p/q be the rational approximation of the polar angle of W expressed as a fraction of a turn, i.e., $W \approx \mathrm{e}^{i 2\pi \frac{p}{q}}$. Then, the modified formula for B can be stated as follows:

$$B = \begin{cases} -p \log 2 + C_1 \log N + C_2, & \text{if } q \geq N, \\ S_3 - \max(S_1, S_2) + C_3 \log N + C_4, & \text{if } q < N, \end{cases} \quad (64)$$

where C_3 and C_4 are implementation-dependent constants. For calculations with both double and quadruple floating-point precision, our experiments indicate that C_3 can be set to -1 and C_4 to 0.

Figure S13 shows the error prediction obtained using Eq. (21) but with the offset term B defined as in Eq. (64). The results show that the modified formula no longer underestimates the numerical error. That is, the points below zero in Fig. S12c are now in line with all other points.

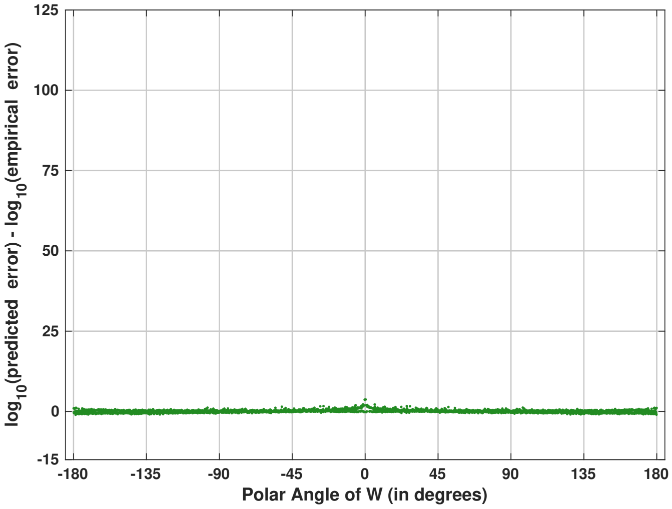

Fig. S13. Visualization of the difference between the logarithms of the predicted and the empirical error, computed using the modified offset term B. This is similar to Fig. S12c, but in this case all predicted errors are close to the empirically-observed errors and all points are plotted in green.
